# Canine Distemper Virus‐Induced Transcriptional Modulation of Canine Histiocytic Sarcoma Cells in an In Vitro Model of Tumour Zones

**DOI:** 10.1111/jcmm.71409

**Published:** 2026-10-14

**Authors:** Thanaporn Asawapattanakul, Klaus Schughart, Robert Geffers, Maren von Köckritz‐Blickwede, Federico Armando, Wolfgang Baumgärtner, Christina Puff

**Affiliations:** ^1^ Department of Pathology University of Veterinary Medicine Hannover Hannover Germany; ^2^ Center for Systems Neuroscience (ZSN) Hannover Germany; ^3^ Institute of Virology Münster University of Münster Münster Germany; ^4^ Department of Microbiology, Immunology and Biochemistry University of Tennessee Health Science Center Memphis Tennessee USA; ^5^ Helmholtz Center for Infection Research Braunschweig Germany; ^6^ Institute of Biochemistry University of Veterinary Medicine Hannover Hannover Germany; ^7^ Research Center for Emerging Infections and Zoonoses (RIZ) Hannover Germany; ^8^ Unit of Pathology, Department of Veterinary Science University of Parma Parma Italy

**Keywords:** canine distemper virus, canine histiocytic sarcoma, DH82, differential gene expression, enrichment analysis, hypoxia, oncolytic virotherapy, starvation, transcriptomics, tumour zones

## Abstract

Canine distemper virus (Onderstepoort strain; CDVOnd) exhibits potential oncolytic properties. However, the detailed molecular mechanisms remain undetermined so far. This study investigated the influence of a CDVOnd infection and various culture conditions on gene expression patterns in canine histiocytic sarcoma (DH82) cells at different time points simulating different zones within a solid tumour. Persistently CDVOnd‐infected DH82 cells and non‐infected controls were cultured under standard conditions, 1% hypoxia, and starvation. Cells were analysed on day 1 (short‐term) and day 3 (prolonged exposure) using proliferation assays and RNA sequencing. The number of infected cells significantly decreased under altered compared to control conditions and decreased after prolonged compared to short‐term exposure. Enrichment analysis revealed that persistent CDV infection, compared to non‐infected controls, upregulated genes associated with immune processes and downregulated tissue developmental pathways, including angiogenesis across all conditions and time points. Compared to infected cells maintained under control conditions, hypoxia and starvation induced distinct, condition‐dependent transcriptomic responses regardless of exposure duration. Hypoxia led to suppression of immune‐related processes and promoted angiogenesis while starvation resulted in an opposite pattern. This suggests that CDV infection induces transcriptomic changes across all zones suggesting anti‐tumour effects, while microenvironmental conditions might affect the efficacy of CDV‐based virotherapy.

## 1 Introduction

Canine histiocytic sarcoma (HS) is a solid malignant tumour in dogs characterized by the proliferation of macrophages or dendritic cells. HS can manifest as a localized or disseminated form [1] and is often linked to a poor prognosis, especially when it affects the central nervous system (CNS) [2]. Dogs with HS affecting the CNS often survive only a few days to several months after initial diagnosis [3]. Pembroke Welsh Corgis and Shetland Sheepdogs are known to be predisposed to primary CNS HS [2]. Current treatment options are limited and involve a combination of surgical excision and chemotherapy or radiation therapy, and supportive care [3, 4]. However, this aggressive tumour, along with tumour hypoxia and starvation, which are common characteristics observed in solid tumours, particularly in the central zone, present crucial therapeutic obstacles [5, 6].

Tumour hypoxia and starvation have an impact on the tumour microenvironment (TME) and can increase tumour aggressiveness by enhancing angiogenesis and altering tumour cell metabolism [6, 7]. Thus, studies targeting these conditions have become one of the most attractive topics [8, 9, 10], which could lead to novel strategies for improving treatment efficacy.

Nowadays, there is a growing interest in exploring novel therapeutic approaches, focusing on understanding the molecular pathways involved in HS and developing targeted therapies that could improve treatment outcomes [11, 12].

Oncolysis represents a promising opportunity for therapeutic strategies, utilizing oncolytic viruses to selectively infect and destroy tumour cells while sparing healthy tissue [13]. This approach eliminates tumour cells by directly lysing them, activating the immune system to detect and attack the neoplastic cells, or altering the tumour microenvironment, which induces inflammatory responses and energy metabolism reprogramming [14, 15, 16].

Canine distemper virus (CDV, *Morbillivirus canis*) is classified within the Morbillivirus genus of the *Paramyxoviridae* family, alongside other viruses such as measles virus [17]. Attenuated measles virus has shown potential for destroying tumour cells in various human tumour types such as ovarian cancer [18]. Interestingly, the use of CDV in oncolytic virotherapy presents a similar opportunity [19]. Especially, the vaccine strain Onderstepoort (CDVOnd) has shown an inhibition of tumour growth and increased intratumoral necrosis in murine xenotransplantation models [14, 20]. Moreover, CDVOnd is able to enhance the immune response through an increased number of tumour‐associated macrophages (TAM), altering the expression of matrix metalloproteinases (MMPs) and their inhibitors [14], while reducing angiogenesis [15, 21], and metastatic potential through decreased cell migration [22, 23].

Unfortunately, little is known about transcriptomic alterations of neoplastic cells after treatment with oncolytic viruses in different surrounding conditions of the TME. This includes local hypoxia and starvation in central areas of the neoplasms. As it is known that tumour cells react differently to treatment approaches depending on the surrounding environment [6, 24] a detailed understanding of the local, post therapeutic alterations is needed to evolve strategies to overcome typical causes for recurrent tumour growth like the survival of tumour stem cells [20, 25].

To broaden the understanding of the effects of therapeutic viral applications, this research aims to investigate the influence of a viral infection of tumour cells in vitro cultured under various conditions mimicking different zones within a solid tumour. The hypothesis is that a therapeutic application of an oncolytic virus and differences in the tumour microenvironment may induce specific transcriptional alterations in solid neoplasms, depending on local conditions such as oxygen and nutrient supply, which may influence therapeutic efficacy.

To test this hypothesis, this study comparatively analysed transcriptomic alterations of persistently CDVOnd infected canine histiocytic sarcoma cells cultured in vitro under normoxic, hypoxic, and starving conditions using RNA sequencing, to simulate different zones within a solid canine HS post virotherapy. These gene expressions were compared to non‐infected controls cultured under the same conditions and cells maintained under standard culture conditions.

## 2 Materials and Methods

### 2.1 Cell and Culture Conditions

#### 2.1.1 Cells

Canine histiocytic sarcoma cells (DH82 cells) were obtained from the European Collection of Authenticated Cell Cultures (ECACC No. 94062922).

Persistently CDV‐Onderstepoort strain (CDVOnd)‐infected DH82 cells (DH82Ondpi cells) were produced as described before [26]. Passage 12 of DH82 cells and passage 143 of DH82Ondpi cells were used in this study. DH82Ondpi cells consistently demonstrated an infection rate exceeding 90% and were used to achieve the strongest possible effects [26].

#### 2.1.2 Culture Conditions

Cells were seeded into 25 cm^2^ flasks at a density of 0.33 × 10^6^ cells/flask and cultured in minimal essential medium (MEM) with Earle's salts (PAA, Cölbe, Germany) supplemented with 10% fetal calf serum (PAA), 1% penicillin/streptomycin (PAA), and 1% non‐essential amino acids (Sigma‐Aldrich, Taufkirchen, Germany) for both infected and non‐infected cells. The cells were maintained for 3 days at 37°C in a humidified atmosphere containing 5% CO_2_, 21% O_2_, ensuring optimal growth conditions as previously described [22]. Following this period, the cells were divided into different experimental groups based on infection and culture conditions. They were exposed to standard, hypoxic, and starving conditions for 1 day (short‐term) and 3 days (prolonged exposure). Under hypoxic conditions, flasks were moved to a chamber with 1% O_2_ (1% O_2_, < 10 mmHg, 5% CO_2_) in a hypoxia glove box (Coy Laboratories, Grass Lake, MI, USA) to maintain a consistently low oxygen level during the culture period. For starvation, the culture medium was removed and replaced with new medium lacking fetal calf serum.

### 2.2 Experimental Design Based on Infection, Culture Conditions, and Exposure Time

The experiment comprised twelve subgroups designed to evaluate the effects of persistent CDV infection and culture conditions on DH82 cellular responses. Non‐infected and persistently CDV‐infected DH82 cells were cultured under standard (C), hypoxic (H), or starving (S) conditions for 1 and 3 days, respectively. Non‐infected DH82 cells cultured under standard conditions for 1 and 3 days served as controls. Each group included six biological replicates. The overall experimental design is summarized in Figure S1.

### 2.3 Cell Proliferation Assay

For determination of cellular proliferation, the number of DH82 and DH82Ondpi cells was manually determined in a haemocytometer using trypan blue at day 1 and day 3 of culturing under the specific conditions as previously described [22]. Subsequently, the obtained numbers were compared to the initial cell numbers seeded at day 0 (0.33 × 10^6^ cells). Six individual flasks (*n* = 6) per experimental condition were evaluated per time point, culture condition, and infection status. Data for non‐infected cells were used from a previous publication using the same data set for comparison [27]. The proliferation data for the already published non‐infected DH82 cells [27] and DH82Ondpi cells used for the present study were generated in parallel under the same conditions using the same media.

### 2.4 RNA Sequencing and Differentially Expressed Gene Analysis

The RNA sequencing workflow followed previously described protocols [27], with minor modifications. Total RNA was extracted from DH82 and DH82Ondpi cells cultured under standard, hypoxic, and starving conditions at two time points, as described previously [26]. Briefly, cells were lysed in TRIzol (Invitrogen, Thermo Fisher Scientific, Langenselbold, Germany), followed by chloroform extraction, isopropanol precipitation, and further purification using the RNeasy Mini Kit (Qiagen, Hilden, Germany) with on‐column DNase digestion (RNase‐Free DNase Kit, Qiagen). RNA quality and integrity were assessed using the Agilent 2100 Bioanalyzer (Agilent Technologies, Waldbronn, Germany). Three biological replicates per infection status, culture condition, and time point were processed for RNA sequencing (RNAseq).

RNAseq libraries were prepared from 100 ng of total RNA using the NEBNext Single Cell/Low Input RNA Library Prep Kit for Illumina (New England Biolabs, Ipswich, MA, USA) and sequenced on an Illumina NovaSeq 6000 system (paired‐end, 2 × 150 bp), generating approximately 7.4 × 10^7^ reads per sample. The reads were quality checked (FastQC, v0.11.9), trimmed (Trimagalore, v0.6.7), and mapped (STAR aligner, v2.5.2b) to the sequences of the canine (ENSEMBL *Canis lupus familiaris* , release 112) and the CDV (GenBank Accession Number AF378705.1) genome. Subsequently, the mapped reads were quantified as gene expression counts (RsubRead, v1.32.4).

Data were normalized, log_2_‐transformed, and differential expression analysis was conducted using DESeq2. Genes with an adjusted *p*‐value < 0.05 (adj. *p* < 0.05) and an absolute log_2_ fold change > 1 were considered differentially expressed genes (DEGs). Visualization, hierarchical clustering, functional enrichment, and overlap analyses were performed using integrated Differential Expression and Pathway (iDEP) (http://bioinformatics.sdstate.edu/idep/) [28], ggplot2 (R, v4.5.0), and InteractiVenn (https://www.interactivenn.net/).

### 2.5 Functional Enrichment Analysis

Filtered DEGs were further processed to investigate Gene ontology (GO) biological process terms using GO FAT database and Kyoto Encyclopedia of Genes and Genomes (KEGG) pathways using the Database for Annotation, Visualization, and Integrated Discovery (DAVID) (version Dec. 2021; Knowledgebase 195 v2023q4) (https://david.ncifcrf.gov/). GO terms and KEGG pathways showing a False Discovery Rate (FDR) below 0.05 were considered statistically significant. Visualization of gene expression patterns and pathway data was performed using iDEP [28], Pathview, and ggplot2 package in R (v4.5.0).

### 2.6 Statistical Analysis

For the assessment of cell proliferation, data were presented as median with ranges and analysed using non‐parametric statistical tests. Friedman test was used to evaluate differences across experimental groups and the Mann–Whitney *U* test was performed to identify specific group differences. Multiple testing correction was applied using the Bonferroni method to ensure the reliability of the findings. A *p*‐value less than 0.05 (*p* < 0.05) was considered statistically significant. All comparisons were performed as two‐tailed, using SPSS v29 (SPSS Inc., Chicago, IL, USA), and graphs were generated using GraphPad Prism (GraphPad Software, La Jolla, CA, USA, v8.0.1).

### 2.7 Comparison Design

To study the effect of different culture conditions on the transcriptomic level, pairwise comparisons were carried out as shown in Table 1. Transcriptomic profiles of CDVOnd‐infected DH82 cells under each condition were compared with those under control culture conditions to mimic the post therapeutic alterations of infected‐tumour cells in each tumour zone (Table 1). Furthermore, transcriptomic profiles of CDVOnd‐infected DH82 cells under each condition were compared to each other. These comparisons were designed to simulate the changes in different tumour zones following an oncolytic virotherapy.

**TABLE 1:** Pairwise comparisons designed for transcriptomic analysis of persistently CDVOnd infected DH82 cells in this study.

Comparison	Condition	Timepoint
DH82Ondpi_C_d1 vs. DH82_C_d1	Control condition, infected vs. non infected	Day 1
DH82Ondpi_C_d3 vs. DH82_C_d3	Control condition, infected vs. non infected	Day 3
DH82Ondpi_H_d1 vs. DH82_H_d1	Hypoxia, infected vs. non infected	Day 1
DH82Ondpi_H_d3 vs. DH82_H_d3	Hypoxia, infected vs. non infected	Day 3
DH82Ondpi_S_d1 vs. DH82_S_d1	Starvation, infected vs. non infected	Day 1
DH82Ondpi_S_d3 vs. DH82_S_d3	Starvation, infected vs. non infected	Day 3
DH82Ondpi_H_d1 vs. DH82Ondpi_C_d1	DH82Ondpi, hypoxia vs. control	Day 1
DH82Ondpi_H_d3 vs. DH82Ondpi_C_d3	DH82Ondpi, hypoxia vs. control	Day 3
DH82Ondpi_S_d1 vs. DH82Ondpi_C_d1	DH82Ondpi, starvation vs. control	Day 1
DH82Ondpi_S_d3 vs. DH82Ondpi_C_d3	DH82Ondpi, starvation vs. control	Day 3
DH82Ondpi_C_d3 vs. DH82Ondpi_C_d1	DH82Ondpi, control	Day 3 vs. 1
DH82Ondpi_H_d3 vs. DH82Ondpi_H_d1	DH82Ondpi, hypoxia	Day 3 vs. 1
DH82Ondpi_S_d3 vs. DH82Ondpi_S_d1	DH82Ondpi, starvation	Day 3 vs. 1

Abbreviations: C, control; d, day; DH82, canine histiocytic sarcoma cells; DH82Ondpi, persistently CDVOnd infected DH82 cells; H, hypoxia; S, starvation.

## 3 Results

### 3.1 Infection and Culture Conditions Influence Cell Proliferation

Data for non‐infected cells were obtained from a previously published study using the same data set [27]. Under standard conditions, the number of DH82Ondpi cells was significantly higher than those of non‐infected cells independent of the time point (*p* < 0.05; Table 2 and Figure 1). Under hypoxia, no significant difference was observed between the number of infected and non‐infected cells at day 1, whereas the number of DH82Ondpi was significantly lower than the number of non‐infected controls at day 3 (*p* < 0.05). Under starvation, the number of DH82Ondpi cells was significantly higher than those of non‐infected cells at day 1 (*p* < 0.05). However, at day 3, the number of DH82Ondpi cells was lower than at day 1 and lacked a significant difference to non‐infected controls (*p* > 0.05).

**TABLE 2:** Cell proliferation assay results.

Condition	Total cell number (×10^6^ cells/T25)			
	Day 1		Day 3	
	Median	Range	Median	Range
DH82 cells, control conditions	6.58	5.50–6.99	6.55	5.75–7.85
DH82 cells, hypoxia	4.28	3.40–5.25	0.88	0.65–1.25
DH82 cells, starvation	5.85	4.65–8.05	6.65	6.00–7.75
DH82Ondpi cells, control conditions	9.65	8.45–10.70	8.28	6.45–9.70
DH82Ondpi cells, hypoxia	5.00	3.70–6.35	0.60	0.35–0.85
DH82Ondpi cells, starvation	7.83	6.90–9.50	5.75	4.30–7.30

**FIGURE 1:**
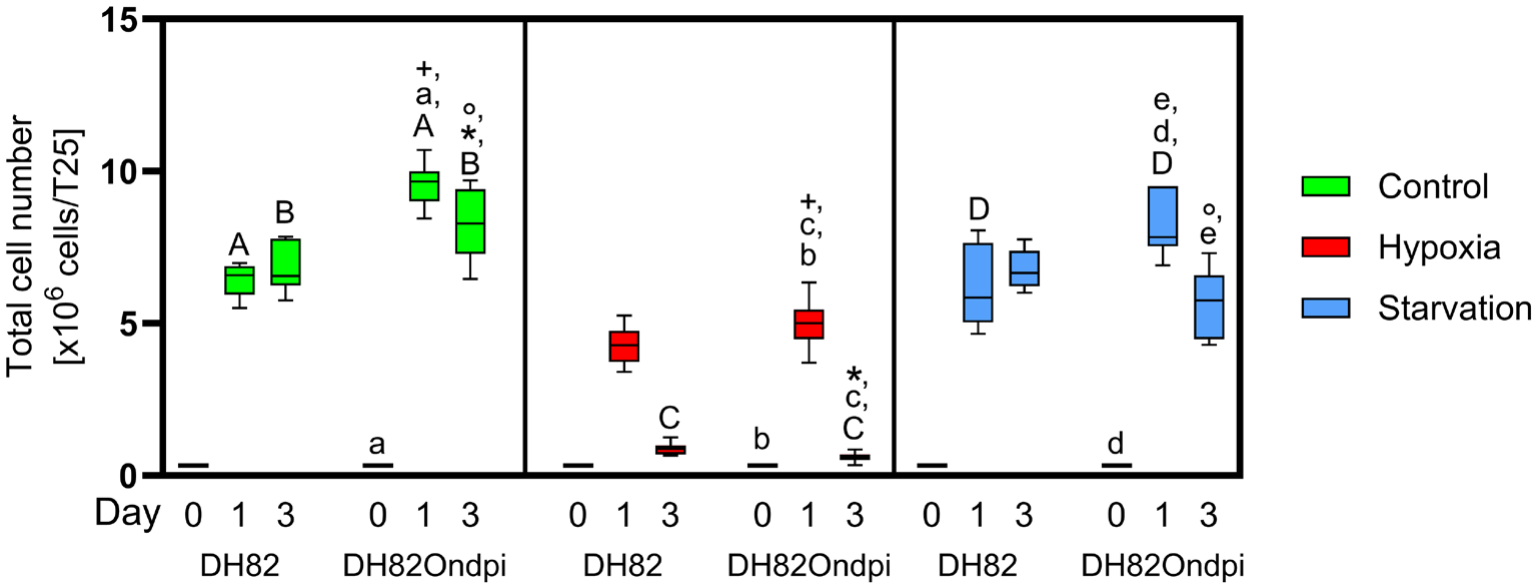
Proliferation assay results for DH82 (non‐infected) and DH82Ondpi (infected) cells cultured under standard, hypoxic, and starving conditions at day 0, day 1, and day 3. Bar graphs represent the median cell number; error bars indicate the range (min–max) of each condition. Same uppercase letter (A–D) indicate significant differences (*p* < 0.05) between infected and non‐infected cells, lowercase letter (a–e) indicate significant differences (*p* < 0.05) between time points, and symbols (+, *, ^○^) indicate significant differences between conditions. Data corresponding to non‐infected cells were referenced from a previously published dataset based on the same experimental condition [27]. CDVOnd, canine distemper virus strain Onderstepoort; DH82, Canine histiocytic sarcoma cells; DH82Ondpi: Persistently CDVOnd‐infected DH82 cells.

To investigate the effect of culture conditions on the growth of DH82Ondpi cells, the number of cells grown under hypoxia and starvation, respectively, was compared to those cultivated under standard conditions. Under hypoxia, the number of DH82Ondpi cells was significantly lower at both time points (day 1 and day 3; *p* < 0.05) compared to DH82Ondpi cells cultured under standard conditions. Under starving conditions, the number of DH82Ondpi cells was significantly lower after prolonged exposure (day 3) compared to DH82Ondpi cells cultured under standard conditions (*p* < 0.05), whereas no significant differences were observed at day 1.

Over time, the number of DH82Ondpi cells significantly increased from day 0 to day 1 independent of the culture condition (*p* < 0.05). From day 1 to day 3, the number of DH82Ondpi cells significantly decreased under hypoxia and starvation, respectively, compared to DH82Ondpi cells cultured under standard conditions (*p* < 0.05).

### 3.2 Persistent CDV Infection Reshapes the Global Transcriptomic Landscape

To investigate the molecular effects of CDV‐based virotherapy of canine HS, transcriptomic profiling was performed on CDV‐infected and non‐infected DH82 cells cultured under standard, hypoxic, and starving conditions for 1 and 3 days. Principal component analysis (PCA) of normalized RNAseq data demonstrated a clear separation between infected and non‐infected cells, with a distinct clustering based on culture condition and duration of exposure. The first two principal components explained 54.3% (PC1) and 14.1% (PC2) of the total variance (Figure 2A).

**FIGURE 2:**
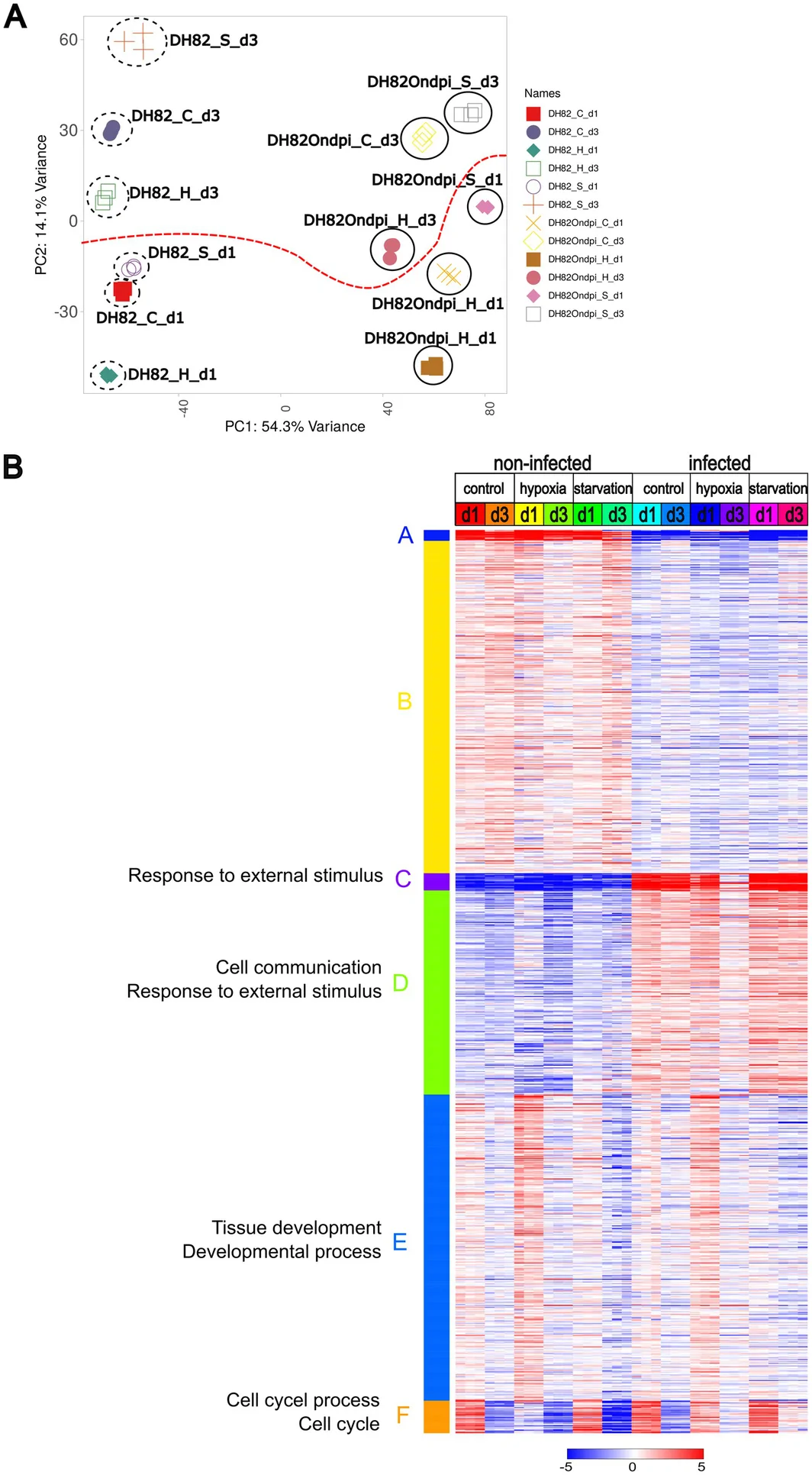
(A) Principal component analysis (PCA) revealed a distinct clustering in the PCA plot, with PC1 (53.7% variance) and PC2 (14.4% variance) distinguishing samples based on infection and time point, respectively. (B) Heatmap with hierarchical clustering shows gene expression patterns in non‐infected and CDVOnd‐infected cells cultured under different conditions. C, control; d1, day 1; d3, day 3; DH82, canine histiocytic sarcoma cells; DH82Ondpi, persistently canine distemper virus strain Onderstepoort‐infected DH82 cells; H, hypoxia; S, starvation.

Transcriptomic changes were assessed by identifying DEGs through pairwise comparisons grouped into three categories: (i) persistently CDVOnd‐infected versus non‐infected DH82 cells at the same time point grown under the same conditions, (ii) CDVOnd‐infected DH82 cells cultured under various conditions versus control conditions at the same time point, and (iii) CDVOnd‐infected DH82 cells cultured under different conditions over time. The number of DEGs varied among comparisons, with CDVOnd‐infected cells showing consistently more DEGs than non‐infected cells (Table 3), particularly under starvation. Volcano plots and the top DEGs are shown in Figure 3, and the top 20 up‐ and downregulated genes for each comparison are listed in Tables S1–S3.

**TABLE 3:** Number of differentially expressed genes^a^ in DH82Ondpi cells cultured under control, hypoxic, and starving conditions at different time points compared to non‐infected controls.

Condition	Timepoint	Upregulated	Downregulated	Total DEGs
Control condition, infected vs. non‐infected	Day 1	1407	1541	2948
Control condition, infected vs. non‐infected	Day 3	1224	1694	2918
Hypoxia, infected vs. non‐infected	Day 1	1429	1655	3084
Hypoxia, infected vs. non‐infected	Day 3	1316	1822	3138
Starvation, infected vs. non‐infected	Day 1	1586	2114	3700
Starvation, infected vs. non‐infected	Day 3	1495	1893	3388

^a^ log2FC > 1, FDR < 0.05.

**FIGURE 3:**
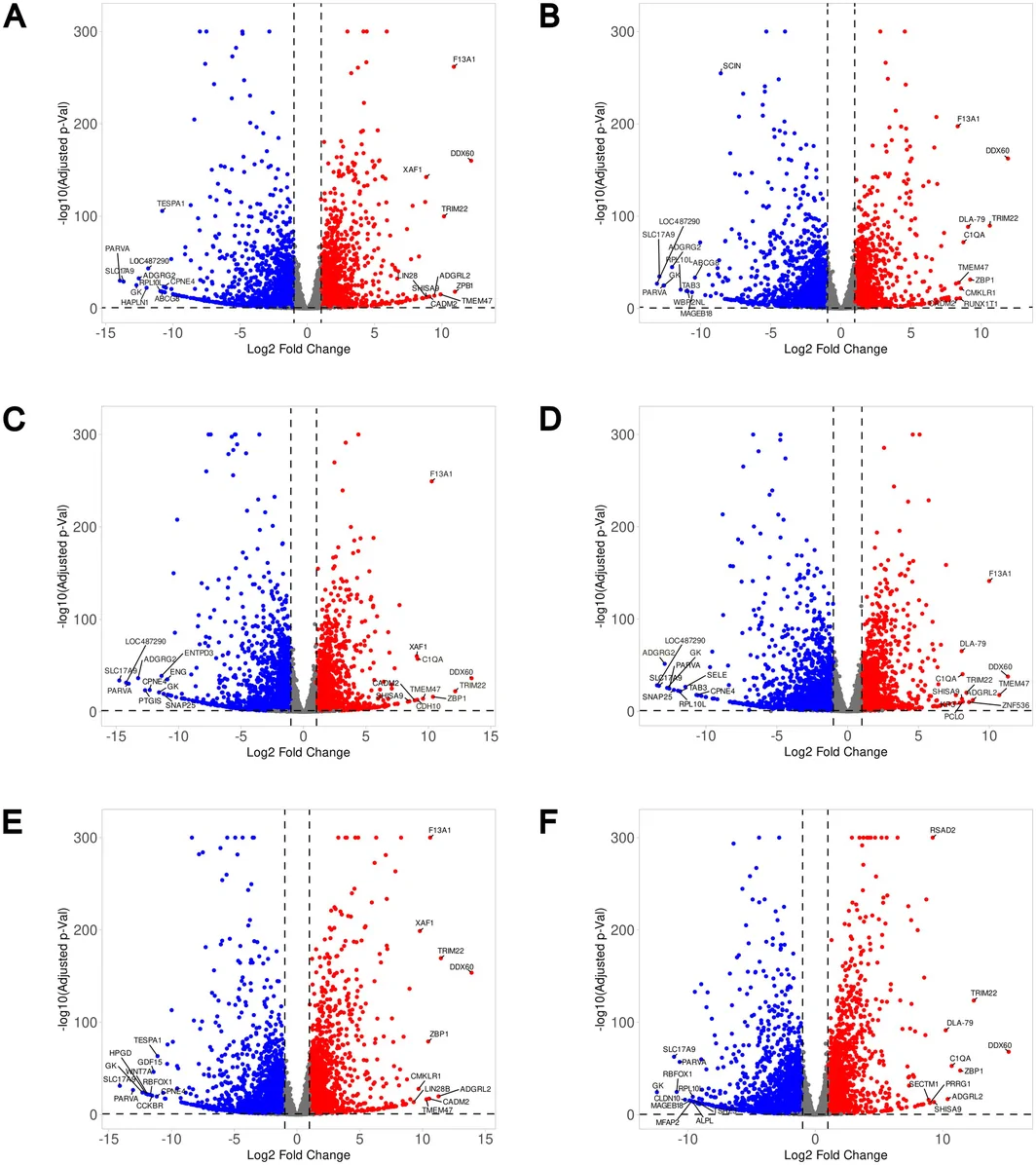
Volcano plots of the gene expression pattern in DH82Ondpi cells cultured under standard (A, B), hypoxic (C, D), and starving (E, F) conditions compared to non‐infected controls at day 1 (A, C, E) and day 3 (B, D, F). DEGs were identified using an adjusted *p*‐value < 0.05 and |log_2_ fold change| > 1 as cutoffs. Significantly up‐ and downregulated genes are shown in red and blue, respectively. The top 10 upregulated and top 10 downregulated DEGs are labelled. C, control; d1, day 1; d3, day 3; DH82, canine histiocytic sarcoma cells; DH82Ondpi, persistently CDVOnd‐infected DH82 cells.

### 3.3 Functional Enrichment Analysis Reveals Broad Transcriptional Reprogramming Under Persistent CDV Infection Compared to Non‐Infected Cells

To investigate the biological functions of the DEGs, GO and KEGG enrichment analyses were performed to identify key biological processes associated with persistent CDV infection. Enrichment results from DAVID are provided in Tables S4–, S6 and visualized in Figures 4, 5, 6 and Table 4.

**FIGURE 4:**
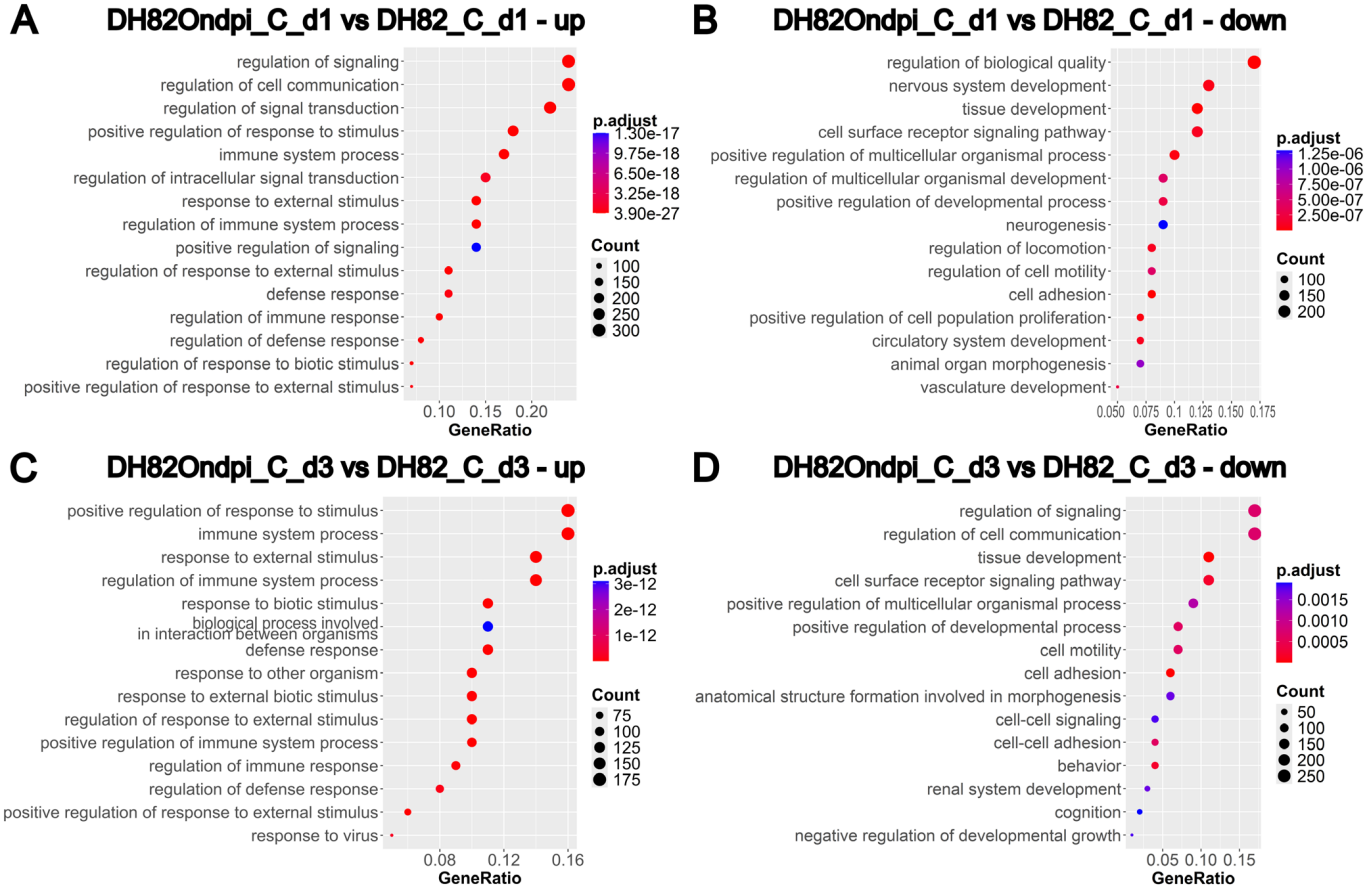
Top 15 enriched gene ontology biological process terms identified from differentially expressed genes (DEGs) in DH82Ondpi cells compared to non‐infected controls under control conditions. (A) Up‐ and (B) down‐regulated terms in short‐term control conditions (d1). (C) Up‐ and (D) down‐regulated terms in prolonged control conditions (d3). Significance was considered using an adjusted *p*‐value < 0.05 and only terms with at least 5 associated genes were included. Bubble colour indicates enrichment significance, and bubble size reflects gene counts. C, control; d1, day 1; d3, day 3; DH82, canine histiocytic sarcoma cells; DH82Ondpi, persistently canine distemper virus strain Onderstepoort‐infected DH82 cells.

**FIGURE 5:**
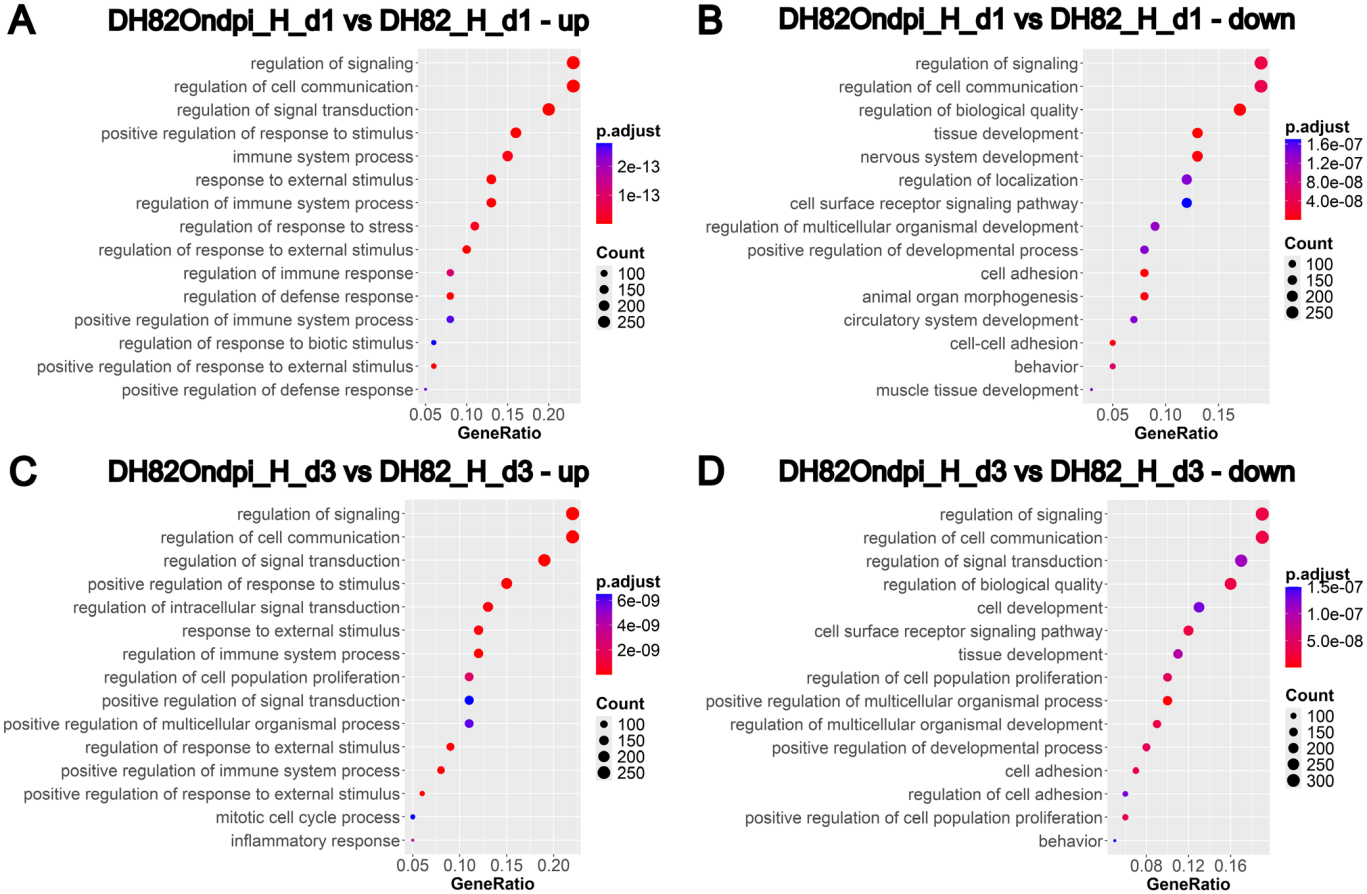
Top 15 enriched gene ontology biological process terms identified from differentially expressed genes (DEGs) in DH82Ondpi cells compared to non‐infected controls under hypoxic conditions. (A) Up‐ and (B) down‐regulated terms in short term hypoxic conditions (d1). (C) Up‐ and (D) down‐regulated terms in prolonged hypoxic conditions (d3). Significance was considered using an adjusted *p*‐value < 0.05 and only terms with at least 5 associated genes were included. Bubble colour indicates enrichment significance, and bubble size reflects gene counts. d1, day 1; d3, day 3; DH82, canine histiocytic sarcoma cells; DH82Ondpi, persistently canine distemper virus strain Onderstepoort‐infected DH82 cells; H, hypoxia.

**FIGURE 6:**
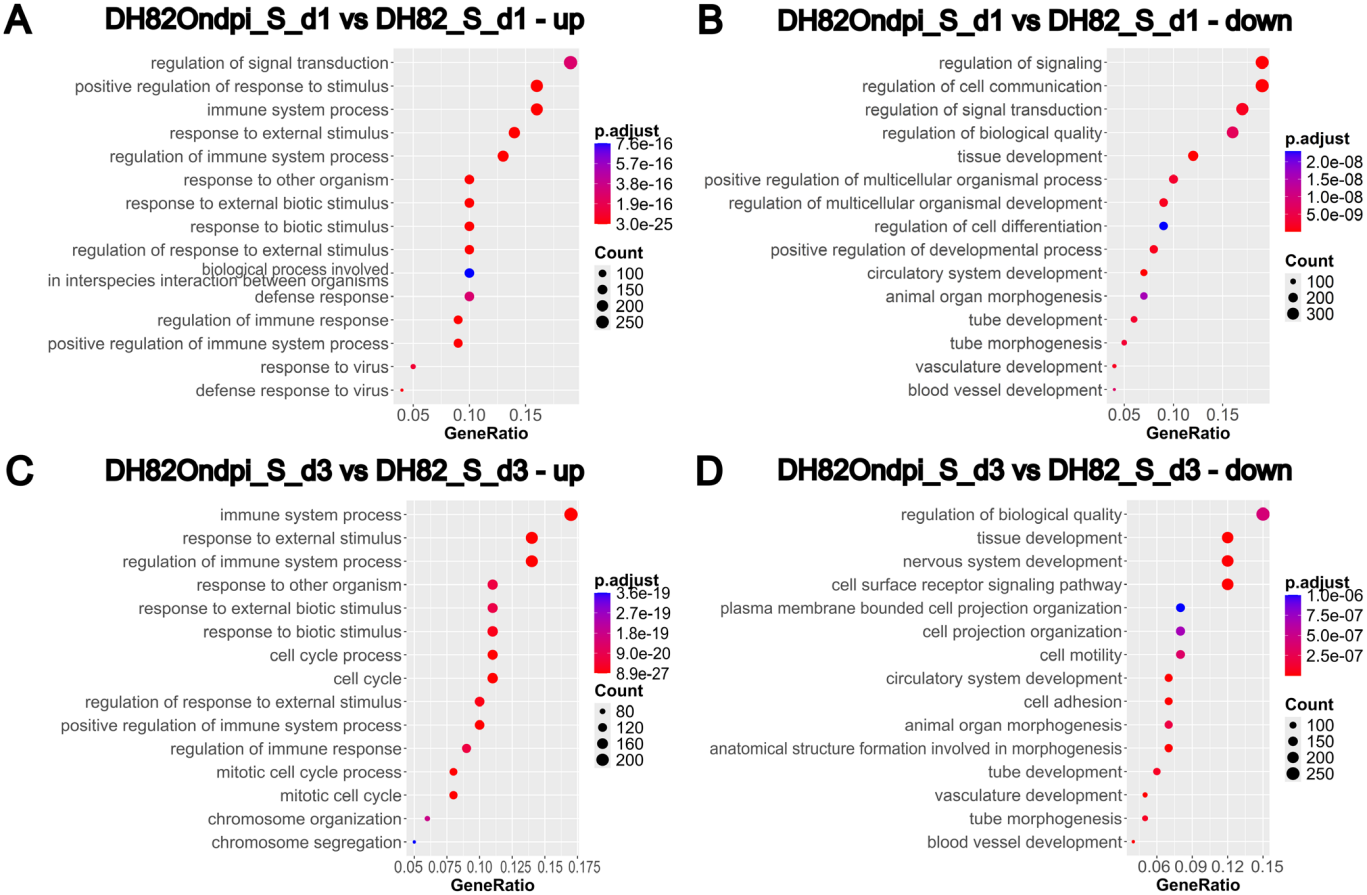
Top 15 enriched gene ontology biological process terms identified from differentially expressed genes (DEGs) in DH82Ondpi cells compared to non‐infected controls under starving conditions. (A) Up‐ and (B) down‐regulated terms in short‐term starving conditions (d1). (C) Up‐ and (D) down‐regulated terms in prolonged starving conditions (d3). Significance was considered using an adjusted *p*‐value < 0.05 and only terms with at least 5 associated genes were included. Bubble colour indicates enrichment significance, and bubble size reflects gene counts. d1, day 1; d3, day 3; DH82, canine histiocytic sarcoma cells; DH82Ondpi, persistently canine distemper virus strain Onderstepoort‐infected DH82 cells; S, starvation.

**TABLE 4:** Differentially expressed genes and functional annotation clusters (KEGG pathways) in DH82Ondpi cells compared to non‐infected controls, ranked by false discovery rate.

Comparisons	Upregulation (FDR value)	Downregulation (FDR value)
Control condition, short‐term (d1) infected vs. non‐infected (total: 2948, up: 1407, down: 1541)	Chemokine signalling pathway (< 0.001), Influenza A (< 0.001), Herpes simplex virus 1 infection (< 0.001), Toll‐like receptor signalling pathway (< 0.001), Cytokine‐cytokine receptor interaction (< 0.001), Measles (< 0.001), Chagas disease (< 0.001), Rap1 signalling pathway (< 0.001), Lipid and atherosclerosis (< 0.001), JAK–STAT signalling pathway (< 0.001)	Cytoskeleton in muscle cells (< 0.001), Pathways in cancer (< 0.05), Calcium signalling pathway (< 0.05), Focal adhesion (< 0.05), PI3K‐Akt signalling pathway (< 0.05), Cytokine‐cytokine receptor interaction (< 0.05), ECM‐receptor interaction (< 0.05), Gastric acid secretion (< 0.05), Ras signalling pathway (< 0.05), Cell adhesion molecules (< 0.05)
Control condition, prolonged (d3) infected vs. non‐infected (total: 2918, up: 1224, down: 1694)	Influenza A (< 0.001), Measles (< 0.001), Chemokine signalling pathway (< 0.001), Herpes simplex virus 1 infection (< 0.001), Toll‐like receptor signalling pathway (< 0.01), Cytokine‐cytokine receptor interaction (< 0.01), RIG‐I‐like receptor signalling pathway (< 0.01), Viral protein interaction with cytokine and cytokine receptor (< 0.01), Complement and coagulation cascades (< 0.01), Cytosolic DNA‐sensing pathway (< 0.05)	Cytoskeleton in muscle cells (< 0.001), Metabolic pathways (< 0.05)
Hypoxia condition, short‐term (d1) infected vs. non‐infected (total: 3084, up: 1429, down: 1655)	Influenza A (< 0.001), Measles (< 0.001), Herpes simplex virus 1 infection (< 0.001), Toll‐like receptor signalling pathway (< 0.001), Cytokine‐cytokine receptor interaction (< 0.001), Viral protein interaction with cytokine and cytokine receptor (< 0.001), Chemokine signalling pathway (< 0.01), RIG‐I‐like receptor signalling pathway (< 0.01), Pertussis (< 0.01), Hepatitis B (< 0.01)	Cytoskeleton in muscle cells (< 0.001), ABC transporters (< 0.01), Hypertrophic cardiomyopathy (< 0.01), Arrhythmogenic right ventricular cardiomyopathy (< 0.01), ECM‐receptor interaction (< 0.01), Focal adhesion (< 0.05), Calcium signalling pathway (< 0.05), PI3K‐Akt signalling pathway (< 0.05), Pathways in cancer (< 0.05)
Hypoxia condition, prolonged (d3) infected vs. non‐infected (total: 3138, up: 1316, down: 1822)	Chemokine signalling pathway (< 0.01), Relaxin signalling pathway (< 0.05), PI3K‐Akt signalling pathway (< 0.05), Cell cycle (< 0.05), Transcriptional misregulation in cancer (< 0.05)	Cytoskeleton in muscle cells (< 0.001), Cell adhesion molecules (< 0.001), NF‐kappa B signalling pathway (< 0.01), Pathways in cancer (< 0.01), Calcium signalling pathway (< 0.05), Cytokine‐cytokine receptor interaction (< 0.05), Viral myocarditis (< 0.05), Allograft rejection (< 0.05)
Starvation condition, short‐term (d1) infected vs. non‐infected (total: 3700, up: 1586, down: 2114)	Herpes simplex virus 1 infection (< 0.001), Influenza A (< 0.001), Chemokine signalling pathway (< 0.001), Toll‐like receptor signalling pathway (< 0.001), Epstein–Barr virus infection (< 0.001), Measles (< 0.001), RIG‐I‐like receptor signalling pathway (< 0.001), Cytokine‐cytokine receptor interaction (< 0.001), Pertussis (< 0.001), Chagas disease (< 0.001)	Metabolic pathways (< 0.01), Focal adhesion (< 0.01), Pathways in cancer (< 0.01), Cytoskeleton in muscle cells (< 0.01), Hippo signalling pathway (< 0.01), Gastric cancer (< 0.01), PI3K‐Akt signalling pathway (< 0.05), Small cell lung cancer (< 0.05), Basal cell carcinoma (< 0.05), Hypertrophic cardiomyopathy (< 0.05)
Starvation condition, prolonged (d3) infected vs. non‐infected (total: 3388, up: 1495, down: 1893)	Cell cycle (< 0.001), Herpes simplex virus 1 infection (< 0.001), Measles (< 0.001), Epstein–Barr virus infection (< 0.001), Influenza A (< 0.01), Cytokine‐cytokine receptor interaction (< 0.01), Cell adhesion molecules (< 0.01), Fanconi anaemia pathway (< 0.01), Chemokine signalling pathway (< 0.01), Cytosolic DNA‐sensing pathway (< 0.01)	Cytoskeleton in muscle cells (< 0.001), Calcium signalling pathway (< 0.001), Metabolic pathways (< 0.01), ABC transporters (< 0.05)

Abbreviations: C, control; d1, day 1; d3, day 3; DH82Ondpi, persistently canine distemper virus‐infected canine histiocytic sarcoma cells; FDR, False Discovery Rate; H, hypoxia; n.s. = no significantly enriched KEGG pathways; S, starvation.

Under control conditions, CDV‐infected cells showed a marked enrichment of GO terms related to immune response, defence response, response to stimulus, and signal transduction compared to non‐infected cells at both timepoints. KEGG pathway analysis supported these findings, revealing a significant upregulation of immune‐ and infection‐related pathways, including chemokine signalling, Toll‐like receptor, JAK–STAT, Rap1, and Nod‐like receptor signalling, as well as pathways associated with viral infections such as influenza, measles, and Herpes simplex virus. In contrast, downregulated GO terms and KEGG pathways were primarily associated with developmental processes, particularly angiogenesis, cell adhesion, cell cycle regulation, metabolic pathways, cell movement and signalling pathways including PI3k‐Akt, and Ras signalling pathways.

Under hypoxic conditions, significant alterations in gene expression profiles of infected cells, compared to non‐infected cells, were observed, which were linked to several processes. Upregulated processes included GO terms associated with immune regulation and cellular responses to stress and stimuli at both time points. KEGG analysis demonstrated enrichment of immune and antiviral pathways, including Toll‐like, RIG‐I‐like, Nod‐like receptor, and TNF signalling pathways, whereas pathways related to extracellular matrix organization, cell adhesion, PI3K–Akt signalling and cancer progression were downregulated. Additionally, prolonged hypoxia upregulated pathways involved in regulation of cell proliferation and mitotic cell cycle processes.

Under starving conditions, the strongest transcriptomic changes in infected cells compared to non‐infected controls were observed after short‐term exposure (total of 3700 DEGs). Upregulated DEGs were predominantly linked to immune response, defence mechanisms, response to virus and cytokine‐related signalling, while downregulated genes were enriched in processes related to new blood vessel formation and cell adhesion. KEGG pathway analysis revealed a strong activation of viral infection pathways and immune signalling. In addition, a suppression of metabolic, adhesion, and cancer‐related pathways was present. These transcriptional patterns continued under prolonged starvation with an additional upregulation of cell cycle–related processes and downregulation of cell motility.

### 3.4 Shared Enriched GO Terms and Overlapping DEGs Across Experimental Conditions Under Persistent CDV Infection

To identify common genes associated with GO terms regulated in infected DH82 cells compared to non‐infected controls independent of the culture condition, overlapping DEGs associated with enriched immune system regulation (upregulation) and circulatory system development (downregulation) were analysed. This analysis identified 63 shared upregulated DEGs and 49 shared downregulated DEGs (Figure 7 and Table S7–, S8).

**FIGURE 7:**
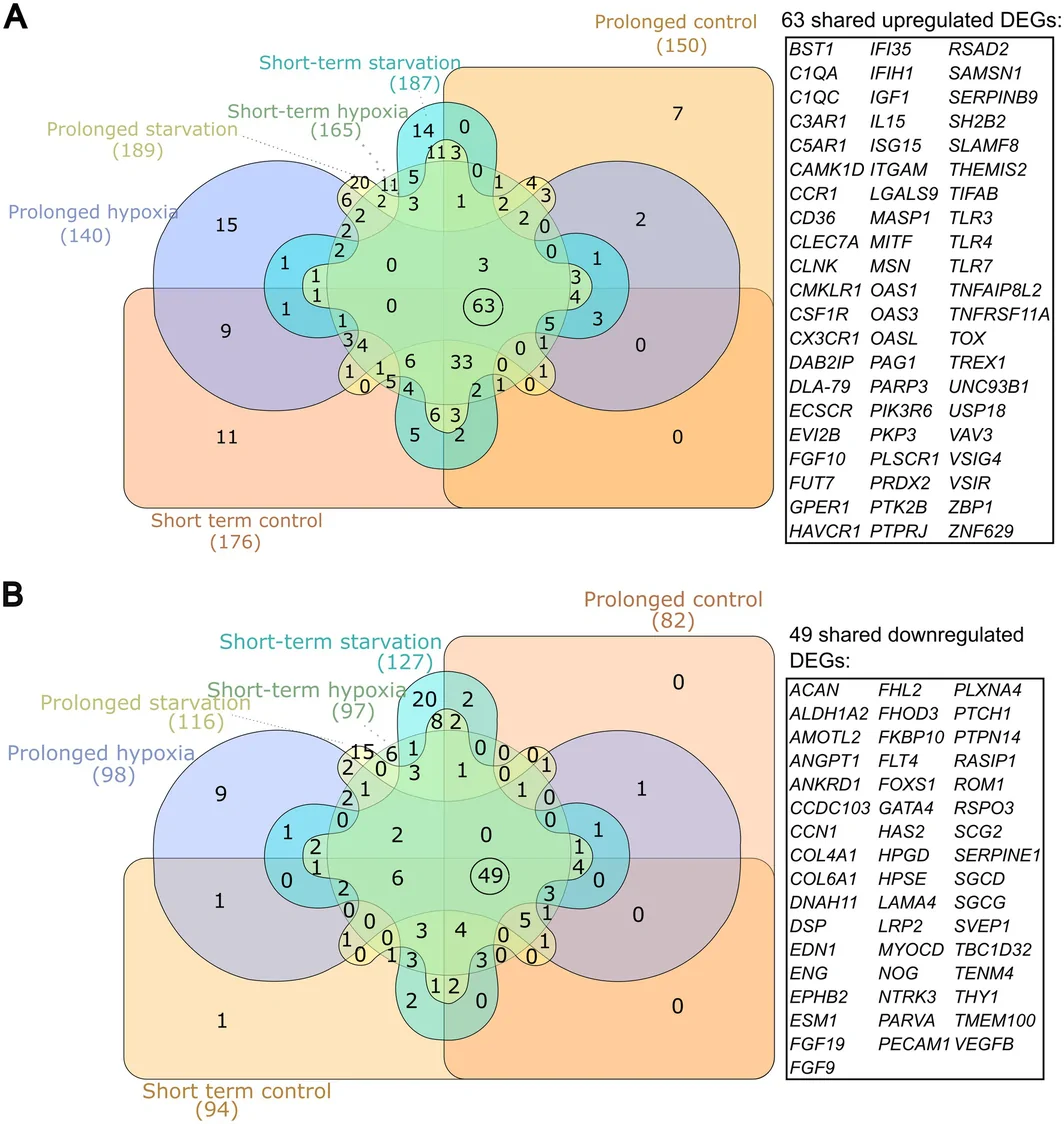
Venn diagram of common DEGs of all conditions compared to non‐infected DH82 cells. (A) Upregulated genes and 63 gene names associated with the GO term regulation of immune system process. (B) Downregulated genes and 49 gene names involved in the GO terms circulatory system development.

### 3.5 Culture Condition‐Dependent Transcriptional Modulation in Persistently Infected Cells Compared to Control Conditions

To evaluate the impact of environmental conditions on CDVOnd infected DH82 cells, the gene expression of cells cultured under hypoxia and starvation, respectively, was compared to infected cells maintained under control conditions (Table 1). Volcano plots and the top DEGs are shown in Figure 8. Hypoxia predominantly resulted in downregulation of immune‐related genes, while upregulated genes were connected to development and angiogenesis after short‐term exposure, and to cell cycle, apoptosis, cell movement, vasculature development and stress response after prolonged exposure. In contrast, short‐term starvation activated genes associated with the immune system and chemotaxis, but suppressed genes linked to development, particularly the circulatory system, and proliferation. Prolonged starvation upregulated genes associated with the cell cycle, immune system process, response to stress and metabolic process while downregulated genes were linked to tissue and circulatory system development, and cell adhesion (Figures 9, 10, 11 and Table 5).

**FIGURE 8:**
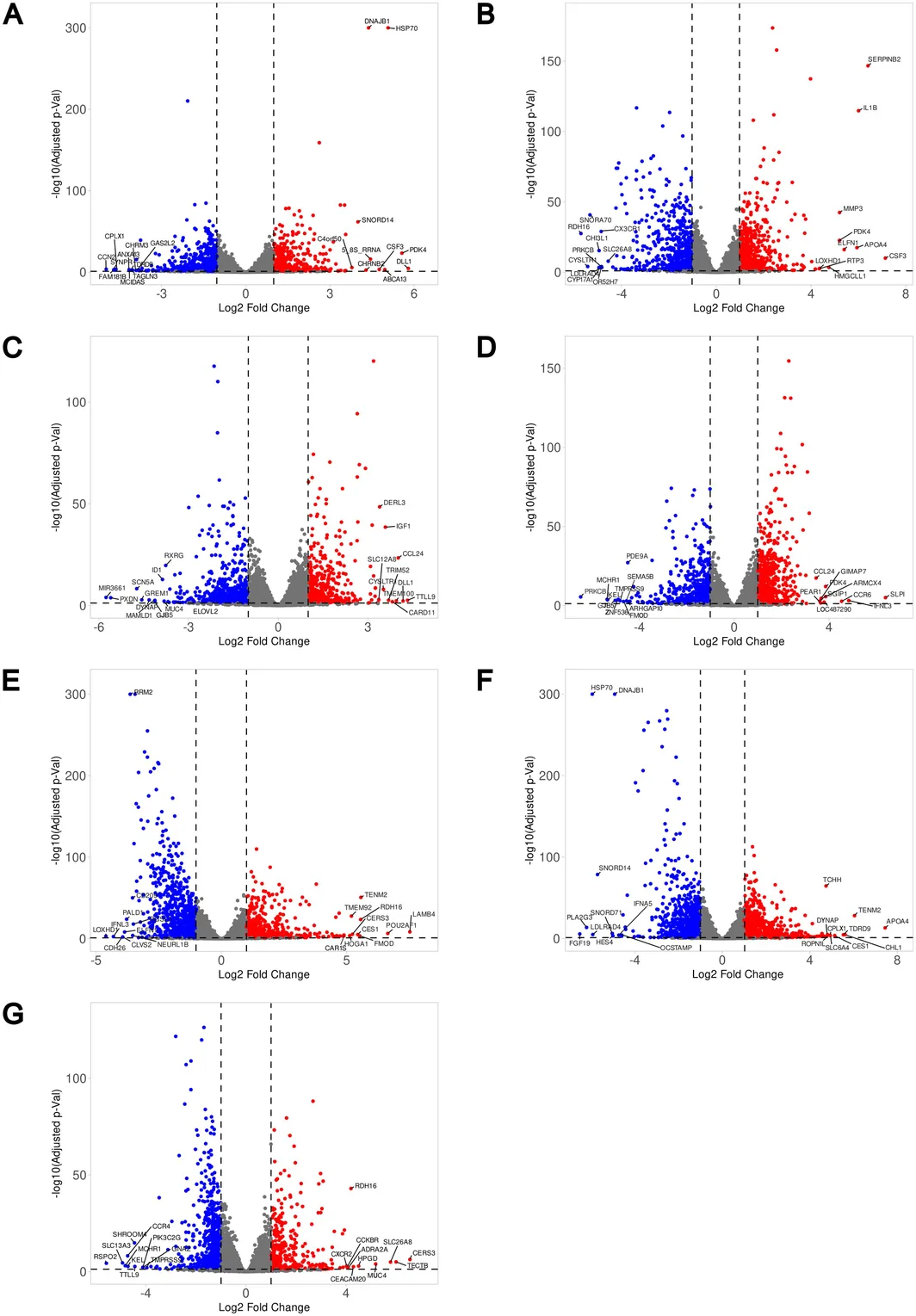
Volcano plots (A–D) of the gene expression pattern in DH82Ondpi cells cultured under hypoxia (A, B) at day 1 (A; short term exposure) and day 3 (B; prolonged exposure) and under starvation (C, D) at day 1 (C; short term exposure) and day 3 (D; prolonged exposure), compared to control conditions. Volcano plots (E–G) of DEGs in DH82Ondpi cells cultured under standard (E), hypoxia (F), and starvation (G) after prolonged exposure (3d) compared to a short time exposure (1d). DEGs were identified using an adjusted *p*‐value < 0.05 and |log_2_ fold change| > 1| as cutoffs. Significantly up‐ and downregulated genes are shown in red and blue, respectively. The top 10 upregulated and top 10 downregulated DEGs are labelled. C, control; d1, day 1; d3, day 3; DH82, canine histiocytic sarcoma cells; DH82Ondpi, persistently CDVOnd‐infected DH82 cells.

**FIGURE 9:**
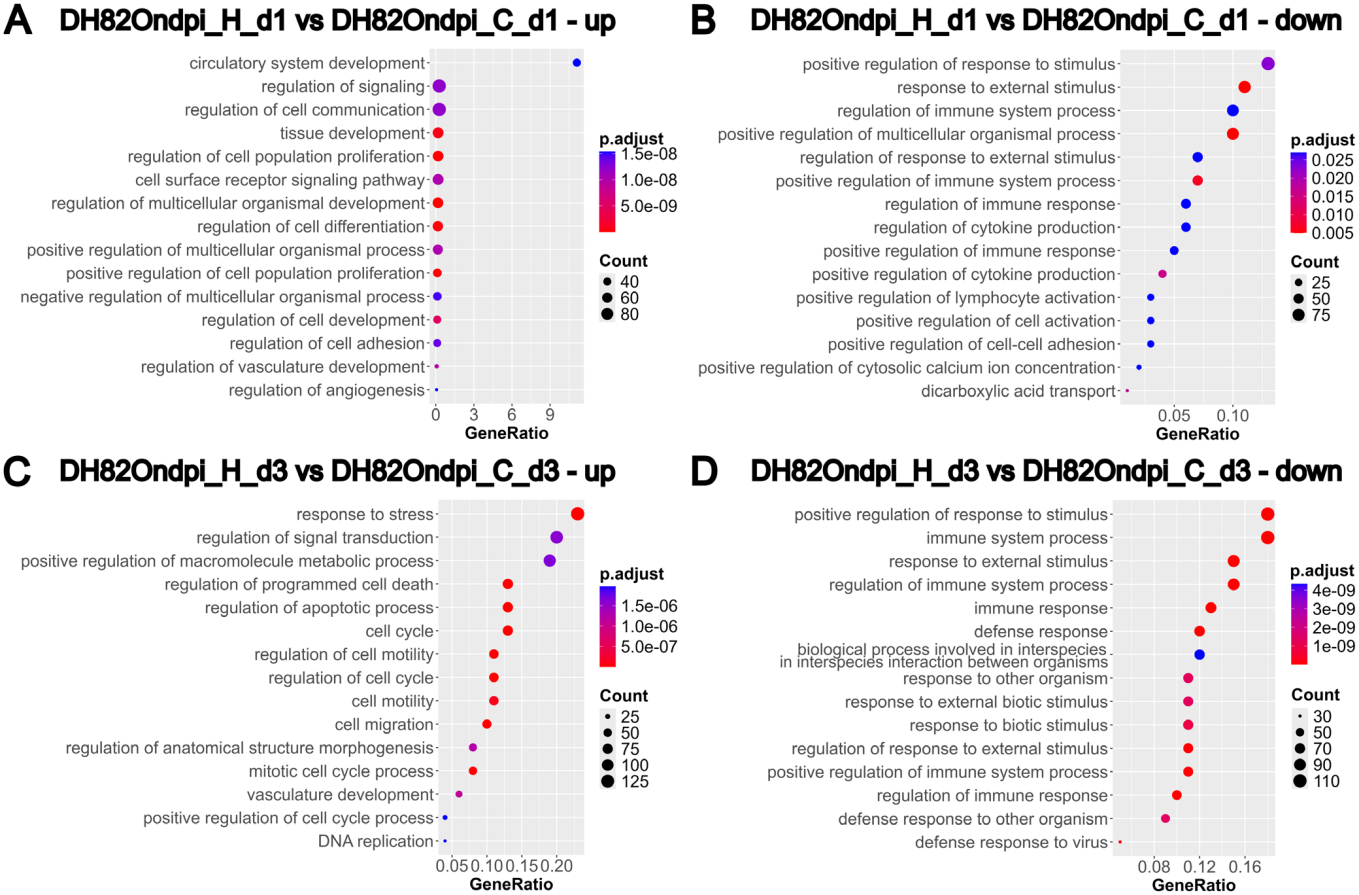
Top 15 enriched gene ontology biological process terms identified from differentially expressed genes in DH82Ondpi cells under hypoxic conditions compared to control conditions. (A) Up‐ and (B) down‐regulated terms in short term hypoxia (d1). (C) Up‐ and (D) down‐regulated terms in prolonged hypoxia (d3). Significance was considered using an adjusted *p*‐value < 0.05, and only terms with at least 5 associated genes were included. Bubble colour indicates enrichment significance, and bubble size reflects gene counts. C, control; d1, day 1; d3, day 3; DH82, canine histiocytic sarcoma cells; DH82Ondpi, persistently canine distemper virus strain Onderstepoort‐infected DH82 cells; H, hypoxia.

**FIGURE 10:**
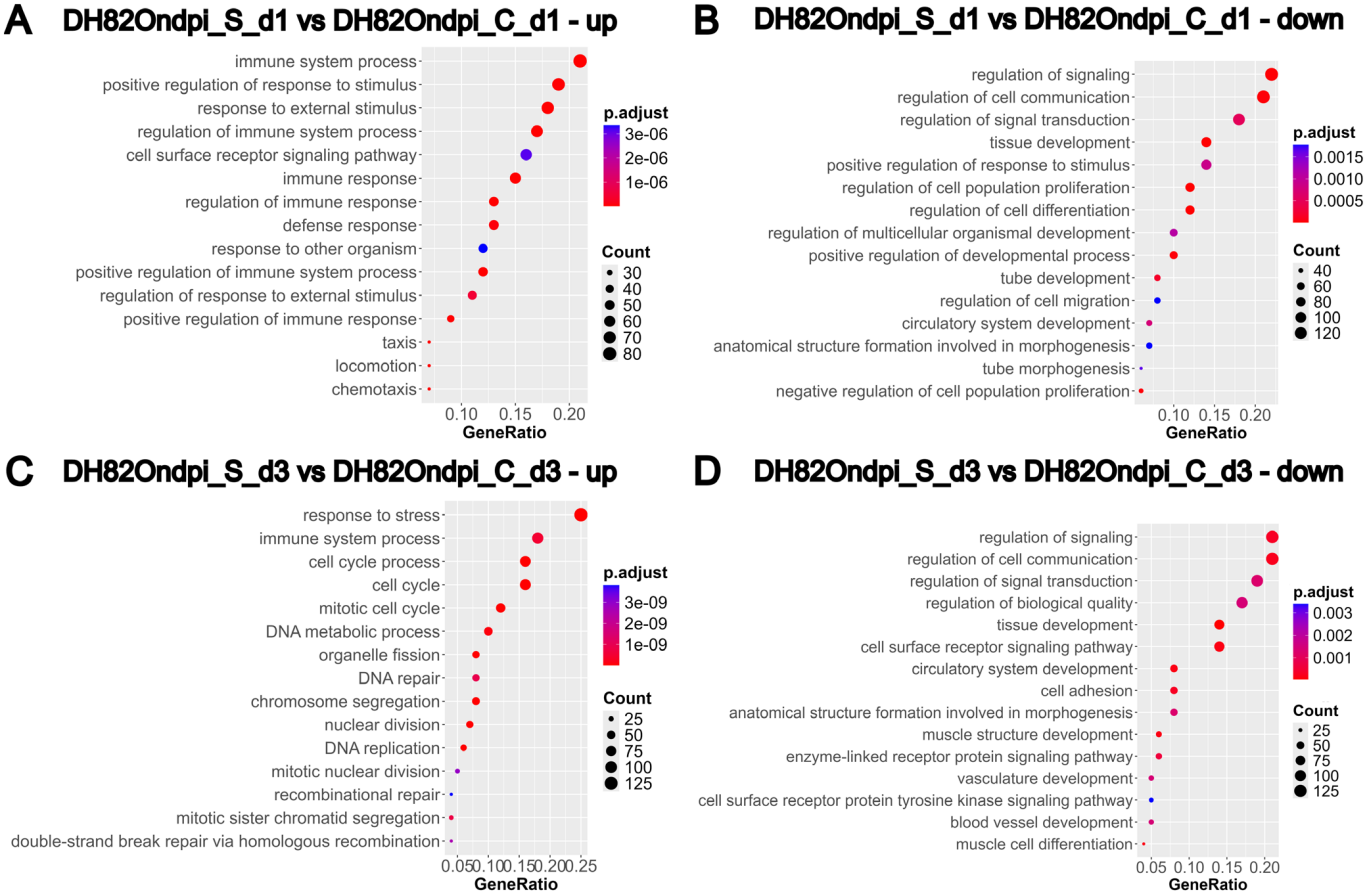
Top 15 enriched gene ontology biological process terms identified from differentially expressed genes in DH82Ondpi cells under starving conditions compared to control conditions. (A) Up‐ and (B) down‐regulated terms in short term starvation (d1). (C) Up‐ and (D) down‐regulated terms in prolonged starvation (d3). Significance was considered using an adjusted *p*‐value < 0.05, and only terms with at least 5 associated genes were included. Bubble colour indicates enrichment significance, and bubble size reflects gene counts. C, control; d1, day 1; d3, day 3; DH82, canine histiocytic sarcoma cells; DH82Ondpi, persistently canine distemper virus strain Onderstepoort‐infected DH82 cells; S, starvation.

**FIGURE 11:**
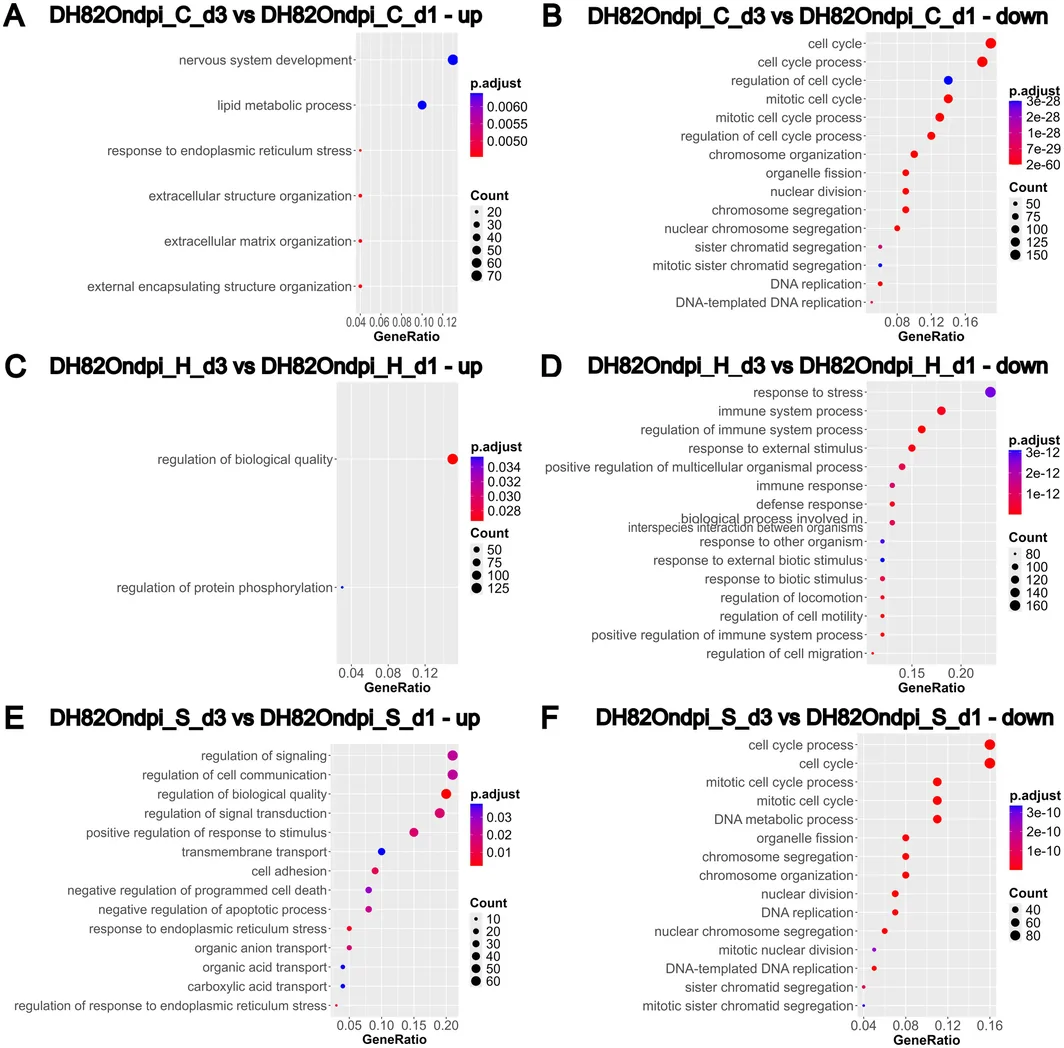
Top 15 enriched gene ontology biological process terms identified from differentially expressed genes in DH82Ondpi cells after prolonged culture compared to short term culture under various conditions. (A) Up‐ and (B) down‐regulated terms in control conditions over time. (C) Up‐ and (D) down‐regulated terms in hypoxia over time. (E) Up‐ and (F) down‐regulated terms in starvation over time. Significance was considered using an adjusted *p*‐value < 0.05, and only terms with at least 5 associated genes were included. Bubble colour indicates enrichment significance, and bubble size reflects gene counts. C, control; d1, day 1; d3, day 3; DH82, canine histiocytic sarcoma cells; DH82Ondpi, persistently canine distemper virus strain Onderstepoort‐infected DH82 cells; H, hypoxia; S, starvation.

**TABLE 5:** Differentially expressed genes and functional annotation clusters (KEGG pathways) in DH82Ondpi cells cultured under various conditions compared to control conditions ranked by false discovery rate.

Comparisons	Upregulation (FDR value)	Downregulation (FDR value)
Short‐term hypoxia (1d) (total: 1068, Up: 372, Down: 696)	TNF signalling pathway (< 0.001), pathways in cancer (< 0.001), PI3K‐Akt signalling pathway (< 0.001), NF‐kappa B signalling pathway (< 0.001), MAPK signalling pathway (< 0.001), breast cancer (< 0.001), IL‐17 signalling pathway (< 0.001), alcoholic liver disease (< 0.001), cytoskeleton in muscle cells (< 0.001), JAK–STAT signalling pathway (< 0.001)	n.s.
Short‐term starvation (1d) (total: 1028, Up: 364, Down: 664)	Cytokine‐cytokine receptor interaction (< 0.001), Epstein–Barr virus infection (< 0.001), viral protein interaction with cytokine and cytokine receptor (< 0.001), Complement and coagulation cascades (< 0.05), Allograft rejection (< 0.05), Type I diabetes mellitus (< 0.05), cell adhesion molecules (< 0.05)	Negative regulation of cell population proliferation (< 0.001)
Prolonged hypoxia (3d) (total: 1102, Up: 551, Down: 551)	Cytokine‐cytokine receptor interaction (< 0.001), cell cycle (< 0.001), IL‐17 signalling pathway (< 0.001), malaria (< 0.001), TNF signalling pathway (< 0.001), rheumatoid arthritis (< 0.001), cellular senescence (< 0.001), pathways in cancer (< 0.01), NF‐kappa B signalling pathway (< 0.01), viral protein interaction with cytokine and cytokine receptor (< 0.01)	Herpes simplex virus 1 infection (< 0.001), Epstein–Barr virus infection (< 0.001), Influenza A (< 0.01), calcium signalling pathway (< 0.01), alcoholic liver disease (< 0.01), NOD‐like receptor signalling pathway (< 0.01), antigen processing and presentation (< 0.01), viral protein interaction with cytokine and cytokine receptor (< 0.01), measles (< 0.01), viral myocarditis (< 0.01)
Prolonged starvation (3d) (total: 1084) Up: 514Down: 570	Cell cycle (< 0.001), DNA replication (< 0.001), cytokine‐cytokine receptor interaction (< 0.001), Fanconi anaemia pathway (< 0.01), viral protein interaction with cytokine and cytokine receptor (< 0.05), homologous recombination (< 0.05), complement and coagulation cascades (< 0.05)	Central carbon metabolism in cancer (< 0.05), pathways in cancer (< 0.05), Ras signalling pathway (< 0.05)
Control over time (total: 1322) Up: 480 Down: 842	n.s.	Cell cycle (< 0.001), DNA replication (< 0.001), Fanconi anaemia pathway (< 0.001), mismatch repair (< 0.001), viral protein interaction with cytokine and cytokine receptor (< 0.001), oocyte meiosis (< 0.001), base excision repair (< 0.01), steroid biosynthesis (< 0.01), homologous recombination (< 0.01), cellular senescence (< 0.01)
Hypoxia over time (total: 1555) Up: 832 Down: 723	n.s.	Steroid biosynthesis (< 0.001), chemokine signalling pathway (< 0.001), Epstein–Barr virus infection (< 0.001), complement and coagulation cascades (< 0.001), haematopoietic cell lineage (< 0.001), Herpes simplex virus 1 infection (< 0.01), viral protein interaction with cytokine and cytokine receptor (< 0.01), Pertussis (< 0.01), Toll‐like receptor signalling pathway (< 0.01), cell adhesion molecules (< 0.01)
Starvation over time (total: 849) Up: 296 Down: 553	n.s.	Steroid biosynthesis (< 0.001), cell cycle (< 0.001), DNA replication (< 0.001), Fanconi anaemia pathway (< 0.001), cytokine‐cytokine receptor interaction (< 0.05), homologous recombination (< 0.05), viral protein interaction with cytokine and cytokine receptor (< 0.05)

Abbreviations: C, control; d1, day 1; d3, day 3; DH82Ondpi, persistently canine distemper virus‐infected canine histiocytic sarcoma cells; FDR, False Discovery Rate; H, hypoxia; n.s., no significantly enriched KEGG pathways; S, starvation.

## 4 Discussion

Canine distemper virus (CDV) has shown promising oncolytic potential and has been explored as an alternative tumour treatment [14, 15, 20]. This study focuses on transcriptomic changes in persistently CDV‐infected canine histiocytic sarcoma cells (DH82) cultured under various conditions to model virotherapy responses across distinct areas within a solid tumour. Comparisons between infected and non‐infected cells under the same conditions simulated virotherapy effects on non‐infected tumour cells, whereas comparisons between infected cells across culture conditions reflected zone‐specific adaptation after virotherapy, including potential resistance. In this study, persistently CDV‐infected DH82 cells with infection rates exceeding 90% were utilized to ensure the maximum effect of CDV infection [26]. Short‐ and prolonged exposure to modified culture conditions were designed to simulate intermediate and central tumour regions, respectively, while standard conditions reflected the tumour periphery. This approach is conceptually consistent with previous studies that simulated tumour microenvironment‐like conditions of solid tumours to investigate the effects of hypoxia, nutrient deprivation, and increased acidity on pancreatic tumour cell metabolism and behaviour or transcriptomic alterations in canine histiocytic sarcoma cells [24, 27].

The impact of oncolytic virotherapy on different zones of canine HS in vitro, compared to non‐infected cells indicated that CDV induced obvious transcriptomic alterations with both culture conditions and duration of exposure significantly influencing gene expression profiles. Persistently CDV‐infected DH82 cells exhibited more DEGs than non‐infected cells, particularly under starvation with the highest number observed during short‐term starvation. These findings suggest that CDV infection might increase cellular sensitivity to environmental stress, leading to enhanced transcriptional responses. The pronounced response to short‐term starvation indicates a rapid adaptive capacity to nutrient deprivation in CDV‐infected neoplastic cells, a response not observed in non‐infected DH82 cells [27]. Similar responses have been observed in other cancer models. For example, short‐term starvation has been shown to enhance the sensitivity of breast cancer cell lines to chemotherapy [29], and colorectal carcinoma cell lines to oncolytic measles virus [10].

Functional enrichment revealed that upregulated genes in infected compared to non‐infected DH82 cells were under all conditions (control, hypoxia, and starvation) at all time points mainly involved in biological processes related to immune processes including defence response, response to stimuli, and pro‐inflammatory responses, particularly pathogen associated molecular pattern (PAMP) pathways such as Toll‐like receptor (TLR), Nod‐like receptor (NLR), and RIG‐I‐like receptor (RLR) signalling [30]. Obviously, regulation of immune system processes represented one of the most consistently enriched biological processes across all conditions, suggesting a core cellular response to CDV infection independent of the culture conditions. This suggests that CDV infection could enhance the activation of immune pathways in all areas of solid tumours. Consistent with previous findings, immune‐related genes were significantly up‐regulated following CDV infection in DH82 cells across different viral strains [15, 31]. CDV infection modulates cytokine responses, including IL‐18 and IL‐7 signalling, promotes antigen‐specific immunity [19, 32], and influences macrophage‐related immune gene expression, involving both M1 and M2 polarization [15]. Moreover, transplantation of DH82Ondpi cells displayed a spontaneous tumour regression after an initial tumour formation in immunocompromised mice, supporting a potential immunomodulatory effect [20].

In the context of host immune cells recognizing and detecting viral infections and initiating an immune response, pathogen‐recognition receptors (PRRs) including TLRs, RLRs, and NLRs, are crucial components, leading to the activation of the nuclear factor κB (NF‐κB), mitogen‐activated protein kinase (MAPK), and interferon regulatory factors [30]. Similarly, previous studies revealed that one of the significantly involved pathways in CDV infections is the NF‐κB pathway, contributing to innate immune response and antiviral defence [31, 33]. The RLRs family involves DExDH box RNA helicases (DDX/DHX) such as RIG‐I, MDA‐5, and LGP‐2 genes. RIG‐I (*DDX58*) is associated with the recognition of many viruses such as measles and Newcastle disease virus [34]. Moreover, the combination of RIG‐I and MDA‐5 can induce IFN responses in human measles virus infection [35]. Based on these observations, pathway enrichment analyses revealed that most up‐regulated genes in this study were involved in RIG‐I and TLR signalling pathways, indicating a DH82 cell response to CDV infection through PAMP‐mediated pathways. Obviously, *DDX60* gene, which encodes a DDX/DHX box RNA helicase, was the most upregulated gene across all conditions and likely plays a key role in CDV‐induced antiviral responses, as it has been reported that RIG‐I‐ or MDA5‐dependent expression of Type I IFN was enhanced by *DDX60* expression [36]. In addition, recent studies have also reported that *DDX60* expression is associated with tumour progression and immune signalling pathways in several tumour types such as human pancreatic cancer, which is one of the most aggressive malignancies [37], suggesting that its upregulation may influence both antiviral and tumour‐related cellular processes [36, 37].

Previous studies have demonstrated that oncolytic viruses can inhibit tumour angiogenesis, thus enhancing antitumor effects. For example, IL‐12‐expressing herpes simplex virus (HSV) suppresses angiogenesis and reduces tumour volume in a murine squamous cell carcinoma model [38]. Likewise, CDV reduces microvessel density in canine HS xenografts most likely through oxidative stress–induced dysregulation of HIF‐1α as demonstrated in vitro [15, 21]. Consistent with these observations, the present study revealed a significant downregulation of genes associated with circulatory system development in persistently CDV‐infected DH82 cells across all conditions and time points compared to non‐infected controls. Among all genes, parvin alpha (*PARVA*) was the most downregulated gene across all conditions compared to non‐infected controls. *PARVA* has been involved in tumour progression, angiogenesis, and metastasis through integrin‐linked kinase (ILK)‐Akt‐VEGF signalling, including enhanced endothelial tube formation, which reflects the angiogenic potential relevant to vessel development in solid tumours [39, 40]. Moreover, *PARVA* overexpression enhanced angiogenesis and metastasis in a mouse model of pulmonary adenocarcinoma [39]. These data support that a downregulation of *PARVA* might contribute to the anti‐angiogenic effect observed following CDV infection.

Moreover, one of the additional hallmarks of cancer is metabolic reprogramming, which is essential for the regulation of cellular energy supply and is connected to oncogenic pathways [41, 42]. Numerous studies have investigated the dysregulated metabolism of tumour cells, as well as the pathways involved in restricted microenvironments, particularly a hallmark shift to glycolytic metabolism through glucose uptake, lactate production and mitochondrial respiration that support cell survival [41, 42]. Nonetheless, this study demonstrated that CDV infection resulted in a decrease of gene expression in pathways linked to metabolism and cancer including the PI3K‐Akt signalling pathway compared to non‐infected controls. The latter signalling pathway interferes with cell proliferation, survival, metabolism, and angiogenesis across a range of tumours and is utilized for targeted therapy strategies [7, 43]. Previous studies investigated the combination of inhibiting the PI3K‐Akt signalling pathway with oncolytic viruses to enhance the therapeutic efficacy of virotherapies, particularly in glioblastoma and other malignancies such as osteosarcoma [12, 44]. These results collectively suggest that CDV infection exhibits oncolytic effects in all areas of solid tumours by stimulating the immune system, suppressing angiogenesis and adapting metabolic processes and pathways. These findings are in accordance with several studies showing an attenuation of HIF signalling and angiogenic pathways after oncolytic viral infections, including in vitro reovirus infection in human colon and renal carcinoma cells and in vivo adenovirus studies in solid tumour models such as human colon and lung carcinoma [45, 46].

Finally, another considered aspect is that transcriptomic changes in infected cells reflect distinct zone‐specific responses compared to control conditions. In the simulated central zone, the lack of oxygen and nutrients was associated with a decline in cell numbers. However, pathways enriched under hypoxic conditions were linked to upregulated cell movement and vasculature development, together with downregulated immune‐related processes, suggesting adaptive transcriptional remodelling potentially associated with tumour progression [47], while cell death‐related processes were also upregulated. Although hypoxia has been widely reported to suppress tumour cell proliferation [48, 49], prolonged hypoxia may induce adaptive transcriptional responses that prepare cells for survival and later recovery, as demonstrated by increased growth of human colon carcinoma cells after 2 weeks of hypoxia compared to normoxic conditions, indicating long‐term adaptive responses [50]. Furthermore, the observed enrichment of cell cycle‐related processes may reflect adaptive transcriptional responses to hypoxic stress, as hypoxia is known to interact with cell cycle control mechanisms that support cellular survival and maintain replication fidelity rather than directly indicating increased proliferation [51]. In contrast, starvation primarily upregulated cell cycle and immune system processes, while downregulated circulatory system development, likely enhancing the oncolytic efficacy [10, 52]. Although starvation has been associated with reduced proliferation in various cancer cells, the responses are tumour‐type dependent [52, 53, 54]. Notably, pancreatic and colon cancer cell lines, as well as canine histiocytic sarcoma cells (DH82 cells) exhibit resistance to nutrient deprivation during the first 36 h. In contrast, liver carcinoma cells rapidly decline within this timeframe [27, 53]. These findings align with previous reports on stress‐adaptive mechanisms [6, 51, 55, 56] and signalling pathways in response to metabolic stress that regulates downstream cellular responses [57, 58].

In the intermediate zone, similar to the simulated central zone, proliferation‐, immune‐, and angiogenesis‐related processes, as well as pathways in cancer, were dysregulated, indicating that viral oncolytic properties are influenced by microenvironmental conditions. Reduced immune system activation under hypoxic conditions has been described in both non‐tumour tissue and in tumours where hypoxia‐inducible factors contribute to immune escape [47, 59]. This possible immune escape might limit the treatment efficacy of an oncolytic virotherapy. However, on the other hand, hypoxia‐induced necrosis of tumour cells might enhance the antigenic potential of infected neoplastic cells inducing an increased inflammatory cell infiltration. A similar effect has been observed in a subcutaneous murine xenotransplantation of DH82Ondpi cells. In this model, xenotransplanted DH82Ondpi cells initially led to a tumour formation like non‐infected DH82 cell transplants. While the latter showed a progressive growth, the DH82Ondpi xenotransplants completely regressed spontaneously [15]. Histologically, the inflammatory cell infiltrates in DH82Ondpi xenotransplants were much stronger than in non‐infected tumours and were interpreted to favour regression. To enhance this intratumoral infiltration with macrophages, a strain of CDVOnd was genetically engineered to express granulocyte‐macrophage colony stimulating factor (GM‐CSF; CDVOnd^neon/GM‐CSF^) [60]. Unfortunately, the effect of this virus strain on canine histiocytic sarcoma cells has only been characterized in vitro so far. Taken together, this study demonstrates that CDV infection induces transcriptomic alterations depending on culture conditions in canine histiocytic sarcoma cells indicating tumour zone‐specific responses to oncolytic virotherapy in solid tumours. Notably, CDV infection elicited conserved core effects across all simulated tumour zones, including activation of immune responses and suppression of angiogenesis, highlighting its therapeutic potential. In addition, hypoxia could be a main cause of tumour progression following oncolytic virotherapy through a variety of mechanisms [51] including activation of angiogenesis and pathways in cancer progression including transduction signalling pathways that promote tumour progression and metastasis in all zones of solid tumours [47, 51, 58]. However, it must be taken into account that the present study focuses on persistently CDV infected cells, which constantly show more than 90% infected cells to achieve the maximum effect. The persistent infection of the cells could lead to epigenetic and/or transcriptomic changes that might not occur after an acute infection, which limits the transferability of the results to a possible in vivo treatment of spontaneous canine histiocytic sarcomas. Therefore, further studies including ex vivo spontaneous canine histiocytic sarcoma samples with acute infections as an intermediate step are necessary to clarify the underlying mechanisms and identify specific genes that may serve as therapeutic targets or combination approaches to enhance the efficacy of CDV‐based virotherapy in canine histiocytic sarcomas and potentially other malignancies.

## Author Contributions

**Thanaporn Asawapattanakul:** conceptualization, methodology, formal analysis, investigation, data curation, visualization, writing – original draft, writing – review and editing. **Klaus Schughart:** methodology, formal analysis, writing – review and editing. **Robert Geffers:** methodology, validation, formal analysis, investigation, data curation, writing – original draft, writing – review and editing. **Maren von Köckritz‐Blickwede:** methodology, validation, investigation, resources, writing – original draft, writing – review and editing. **Federico Armando:** conceptualization, methodology, investigation, writing – original draft, writing – review and editing. **Wolfgang Baumgärtner:** conceptualization, validation, resources, funding acquisition, project administration, supervision, writing – original draft, writing – review and editing. **Christina Puff:** conceptualization, methodology, validation, formal analysis, investigation, data curation, project administration, supervision, writing – original draft, writing – review and editing.

## Funding

We acknowledge financial support from the Deutsche Forschungsgemeinschaft (DFG, German Research Foundation)—398066876/GRK 2485/2, Research Training Group VIPER. This study was also supported by intramural grants from the Helmholtz Association (Program Infection and Immunity) awarded to Klaus Schughart. Thanaporn Asawapattanakul received a scholarship from the Royal Thai Government.

## Conflicts of Interest

The authors declare no conflicts of interest.

## Supporting information


**Figure S1:** Schematic overview of the experimental design. Canine distemper virus‐infected and non‐infected canine histiocytic sarcoma cells (DH82 cells) were cultured under three conditions (standard (C), hypoxia (H) and starvation (S)) and harvested at two time points (1 day and 3 days).

**Table S1:** The top 20 up‐ and down‐regulated expressed genes in DH82Ondpi cells cultured under standard conditions compared to non‐infected cells at day 1 and day 3.
**Table S2:** The top 20 up‐ and down‐regulated expressed genes in DH82Ondpi cells cultured under hypoxic conditions compared to non‐infected cells at day 1 and day 3.
**Table S3:** The top 20 up‐ and down‐regulated expressed genes in DH82Ondpi cells cultured under starving conditions compared to non‐infected cells at day 1 and day 3.

**Table S4:** Number of significantly enriched GO terms and KEGG pathways in DH82Ondpi cell cultures cultured under hypoxic, starving and control conditions for different time frames.

**Table S5:** Gene ontology terms of significantly differentially expressed genes in DH82Ondpi cells cultured under hypoxic, starving and control conditions for different time frames.

**Table S6:** KEGG pathways of significantly differentially expressed genes in DH82Ondpi cells under hypoxic, starving and control conditions for different time frames.

**Table S7:** The 63 upregulated gene symbols that overlap in the GO terms regulation of immune system process in DH82 cells persistently infected with CDVOnd cultured under standard, hypoxic and starving conditions for short‐ and prolonged exposure, compared to non‐infected DH82 cells.

**Table S8:** The 49 downregulated gene symbols that overlap in the GO terms circular system development in DH82 cells persistently infected with CDVOnd cultured under standard, hypoxic and starving conditions for short‐ and prolonged exposure, compared to non‐infected DH82 cells.

## Data Availability

Raw sequence reads and a normalized expression matrix are available at the GEO public database (GEO), ID: GSE183324.

## References

[1] V. K. Affolter and P. F. Moore , “Localized and Disseminated Histiocytic Sarcoma of Dendritic Cell Origin in Dogs,” Veterinary Pathology 39 (2002): 74–83, 10.1354/vp.39-1-74. 12102221

[2] I. Toyoda , W. Vernau , B. K. Sturges , et al., “Clinicopathological Characteristics of Histiocytic Sarcoma Affecting the Central Nervous System in Dogs,” Journal of Veterinary Internal Medicine 34 (2020): 828–837, 10.1111/jvim.15673. PMC7096655 31919895

[3] N. G. Dervisis , M. Kiupel , Q. Qin , and L. Cesario , “Clinical Prognostic Factors in Canine Histiocytic Sarcoma,” Veterinary and Comparative Oncology 15 (2017): 1171–1180, 10.1111/vco.12252. 27334037

[4] P. Odatzoglou , T. Kearns , C. Pittaway , and J. M. Dobson , “Impact of Palliative‐Intent Radiotherapy With or Without Chemotherapy on Lameness in Flat Coat Retrievers With Localised Periarticular Histiocytic Sarcoma–a Retrospective Cohort, Single Institution Study,” Veterinary and Comparative Oncology 22 (2024): 22–29, 10.1111/vco.12942. 37935625

[5] A. Saint‐Martin , M. Castañeda‐Patlán , and M. Robles‐Flores , “The Role of Hypoxia Inducible Factors in Cancer Resistance,” Journal of Cellular Signaling 2 (2017): 154, 10.4172/2576-1471.1000154.

[6] T. Osawa and M. Shibuya , “Targeting Cancer Cells Resistant to Hypoxia and Nutrient Starvation to Improve Anti‐Angiogeneic Therapy,” Cell Cycle 12 (2013): 2519–2520, 10.4161/cc.25729. PMC3865031 23907114

[7] R. Courtnay , D. C. Ngo , N. Malik , K. Ververis , S. M. Tortorella , and T. C. Karagiannis , “Cancer Metabolism and the Warburg Effect: The Role of HIF‐1 and PI3K,” Molecular Biology Reports 42 (2015): 841–851, 10.1007/s11033-015-3858-x. 25689954

[8] H.‐R. Jin , J. Wang , Z.‐J. Wang , et al., “Lipid Metabolic Reprogramming in Tumor Microenvironment: From Mechanisms to Therapeutics,” Journal of Hematology & Oncology 16 (2023): 103, 10.1186/s13045-023-01498-2. PMC10498649 37700339

[9] J. Yu , Z. Wei , Q. Li , et al., “Advanced Cancer Starvation Therapy by Simultaneous Deprivation of Lactate and Glucose Using a MOF Nanoplatform,” Advancement of Science 8 (2021): 2101467, 10.1002/advs.202101467. PMC8498878 34363341

[10] G. Scheubeck , S. Berchtold , I. Smirnow , A. Schenk , J. Beil , and U. M. Lauer , “Starvation‐Induced Differential Virotherapy Using an Oncolytic Measles Vaccine Virus,” Viruses 11 (2019): 614, 10.3390/v11070614. PMC6669668 31284426

[11] A. I. Engleberg , Y.‐T. Yang , P. Z. Schall , et al., “Transcriptome Analysis of Canine Histiocytic Sarcoma Tumors and Cell Lines Reveals Multiple Targets for Therapy,” Cancers 17 (2025): 954, 10.3390/cancers17060954. PMC11940154 40149290

[12] J. Jiang , W. Wang , W. Xiang , L. Jiang , and Q. Zhou , “The Phosphoinositide 3‐Kinase Inhibitor ZSTK474 Increases the Susceptibility of Osteosarcoma Cells to Oncolytic Vesicular Stomatitis Virus VSVΔ51 via Aggravating Endoplasmic Reticulum Stress,” Bioengineered 12 (2021): 11847–11857, 10.1080/21655979.2021.1999372. PMC8809975 34720036

[13] R. Ma , Z. Li , E. A. Chiocca , M. A. Caligiuri , and J. Yu , “The Emerging Field of Oncolytic Virus‐Based Cancer Immunotherapy,” Trends Cancer 9 (2023): 122–139, 10.1016/j.trecan.2022.10.003. PMC9877109 36402738

[14] F. Armando , A. Fayyad , S. Arms , et al., “Intratumoral Canine Distemper Virus Infection Inhibits Tumor Growth by Modulation of the Tumor Microenvironment in a Murine Xenograft Model of Canine Histiocytic Sarcoma,” International Journal of Molecular Sciences 22 (2021): 3578, 10.3390/ijms22073578. PMC8037597 33808256

[15] V. M. Pfankuche , I. Spitzbarth , S. Lapp , et al., “Reduced Angiogenic Gene Expression in Morbillivirus‐Triggered Oncolysis in a Translational Model for Histiocytic Sarcoma,” Journal of Cellular and Molecular Medicine 21 (2017): 816–830, 10.1111/jcmm.13023. PMC5345635 27860224

[16] D. Xiong , Q. Wang , W.‐M. Wang , and Z.‐J. Sun , “Tuning Cellular Metabolism for Cancer Virotherapy,” Cancer Letters 592 (2024): 216924, 10.1016/j.canlet.2024.216924. 38718886

[17] S. Lapp , V. M. Pfankuche , W. Baumgärtner , and C. Puff , “Viral Oncolysis‐Can Insights From Measles Be Transferred to Canine Distemper Virus?,” Viruses 6 (2014): 2340–2375, 10.3390/v6062340. PMC4074931 24921409

[18] R. Myers , S. Greiner , M. Harvey , et al., “Oncolytic Activities of Approved Mumps and Measles Vaccines for Therapy of Ovarian Cancer,” Cancer Gene Therapy 12 (2005): 593–599, 10.1038/sj.cgt.7700823. 15746945

[19] J. Zhao , Y. Ren , J. Chen , J. Zheng , and D. Sun , “Viral Pathogenesis, Recombinant Vaccines, and Oncolytic Virotherapy: Applications of the Canine Distemper Virus Reverse Genetics System,” Viruses 12 (2020): 339, 10.3390/v12030339. PMC7150803 32244946

[20] M. S. Lombardo , F. Armando , K. Marek , K. Rohn , W. Baumgärtner , and C. Puff , “Persistence of Infectious Canine Distemper Virus in Murine Xenotransplants of Canine Histiocytic Sarcoma Cells After Intratumoral Application,” International Journal of Molecular Sciences 25 (2024): 8297, 10.3390/ijms25158297. PMC11311720 39125874

[21] F. Armando , M. Gambini , A. Corradi , et al., “Oxidative Stress in Canine Histiocytic Sarcoma Cells Induced by an Infection With Canine Distemper Virus Led to a Dysregulation of HIF‐1α Downstream Pathway Resulting in a Reduced Expression of VEGF‐B *In Vitro* ,” Viruses 12 (2020): 200, 10.3390/v12020200. PMC7077254 32054075

[22] F. Armando , M. Gambini , A. Corradi , et al., “Mesenchymal to Epithelial Transition Driven by Canine Distemper Virus Infection of Canine Histiocytic Sarcoma Cells Contributes to a Reduced Cell Motility *In Vitro* ,” Journal of Cellular and Molecular Medicine 24 (2020): 9332–9348, 10.1111/jcmm.15585. PMC7417708 32627957

[23] V. M. Pfankuche , M. Sayed‐Ahmed , V. B. Contioso , et al., “Persistent Morbillivirus Infection Leads to Altered Cortactin Distribution in Histiocytic Sarcoma Cells With Decreased Cellular Migration Capacity,” PLoS One 11 (2016): e0167517, 10.1371/journal.pone.0167517. PMC5135102 27911942

[24] G. L. Gardner and J. A. Stuart , “Tumor Microenvironment‐Like Conditions Alter Pancreatic Cancer Cell Metabolism and Behavior,” American Journal of Physiology. Cell Physiology 327 (2024): C959–C978, 10.1152/ajpcell.00452.2024. 39183564

[25] M. Najafi , B. Farhood , and K. Mortezaee , “Cancer Stem Cells (CSCs) in Cancer Progression and Therapy,” Journal of Cellular Physiology 234 (2019): 8381–8395, 10.1002/jcp.27740. 30417375

[26] C. Puff , C. Krudewig , I. Imbschweiler , W. Baumgärtner , and S. Alldinger , “Influence of Persistent Canine Distemper Virus Infection on Expression of RECK, Matrix‐Metalloproteinases and Their Inhibitors in a Canine Macrophage/Monocytic Tumour Cell Line (DH82),” Veterinary Journal 182 (2009): 100–107, 10.1016/j.tvjl.2008.03.026. 18684651

[27] T. Asawapattanakul , K. Schughart , M. von Köckritz‐Blickwede , et al., “Transcriptomic Alterations of Canine Histiocytic Sarcoma Cells in Response to Different Stressors,” International Journal of Molecular Sciences 26 (2025): 6629, 10.3390/ijms26146629. PMC12294338 40724877

[28] S. X. Ge , E. W. Son , and R. Yao , “iDEP: An Integrated Web Application for Differential Expression and Pathway Analysis of RNA‐Seq Data,” BMC Bioinformatics 19 (2018): 534, 10.1186/s12859-018-2486-6. PMC6299935 30567491

[29] I. S. Pateras , C. Williams , D. D. Gianniou , et al., “Short Term Starvation Potentiates the Efficacy of Chemotherapy in Triple Negative Breast Cancer via Metabolic Reprogramming,” Journal of Translational Medicine 21 (2023): 169, 10.1186/s12967-023-03935-9. PMC9983166 36869333

[30] K. Veklich , “Pattern‐Recognizing Receptors and the Innate Immune Response to Viral Infection,” Journal of V. N. Karazin Kharkiv National University, Series Medicine 36 (2018): 96–102, 10.26565/2313-6693-2018-36-13.

[31] X. Zheng , Y. Zhu , Z. Zhao , et al., “RNA Sequencing Analyses of Gene Expressions in a Canine Macrophages Cell Line DH82 Infected With Canine Distemper Virus,” Infection, Genetics and Evolution 80 (2020): 104206, 10.1016/j.meegid.2020.104206. 31982604

[32] C. Chen , M. Zhou , X.‐g. Yan , et al., “A Recombinant Canine Distemper Virus Expressing Interleukin‐7 Enhances Humoral Immunity,” Journal of General Virology 100 (2019): 602–615, 10.1099/jgv.0.001247. 30875282

[33] M. Tong , L. Yi , N. Sun , et al., “Quantitative Analysis of Cellular Proteome Alterations in CDV‐Infected Mink Lung Epithelial Cells,” Frontiers in Microbiology 8 (2017): 2564, 10.3389/fmicb.2017.02564. PMC5743685 29312244

[34] P. M. Barral , D. Sarkar , Z.‐z. Su , et al., “Functions of the Cytoplasmic RNA Sensors RIG‐I and MDA‐5: Key Regulators of Innate Immunity,” Pharmacology & Therapeutics 124 (2009): 219–234, 10.1016/j.pharmthera.2009.06.012. PMC3165056 19615405

[35] S. Ikegame , M. Takeda , S. Ohno , Y. Nakatsu , Y. Nakanishi , and Y. Yanagi , “Both RIG‐I and MDA5 RNA Helicases Contribute to the Induction of Alpha/Beta Interferon in Measles Virus‐Infected Human Cells,” Journal of Virology 84 (2010): 372–379, 10.1128/JVI.01690-09. PMC2798399 19846522

[36] M. Miyashita , H. Oshiumi , M. Matsumoto , and T. Seya , “DDX60, a DEXD/H Box Helicase, Is a Novel Antiviral Factor Promoting RIG‐I‐Like Receptor‐Mediated Signaling,” Molecular and Cellular Biology 31 (2011): 3802–3819, 10.1128/MCB.01368-10. PMC3165724 21791617

[37] D. Zhang , E. Zhang , Y. Cai , et al., “Deciphering the Potential Ability of DExD/H‐Box Helicase 60 (DDX60) on the Proliferation, Diagnostic and Prognostic Biomarker in Pancreatic Cancer: A Research Based on Silico, RNA‐Seq and Molecular Biology Experiment,” Hereditas 162 (2025): 6, 10.1186/s41065-024-00361-9. PMC11753068 39844327

[38] R. J. Wong , M.‐K. Chan , Z. Yu , et al., “Angiogenesis Inhibition by an Oncolytic Herpes Virus Expressing Interleukin 12,” Clinical Cancer Research 10 (2004): 4509–4516, 10.1158/1078-0432.CCR-04-0081. 15240543

[39] A.‐H. Huang , S.‐H. Pan , W.‐H. Chang , Q.‐S. Hong , J. J. W. Chen , and S.‐L. Yu , “PARVA Promotes Metastasis by Modulating ILK Signalling Pathway in Lung Adenocarcinoma,” PLoS One 10 (2015): e0118530, 10.1371/journal.pone.0118530. PMC4349696 25738875

[40] M. Takeda , H. Ito , K. Kitahata , et al., “α‐Parvin Expression in Breast Cancer Tissues: Correlation With Clinical Parameters and Prognostic Significance,” Cells 13 (2024): 1572, 10.3390/cells13181572. PMC11430769 39329755

[41] R. M. Pascale , D. F. Calvisi , M. M. Simile , C. F. Feo , and F. Feo , “The Warburg Effect 97 Years After Its Discovery,” Cancers 12 (2020): 2819, 10.3390/cancers12102819. PMC7599761 33008042

[42] P. S. Ward and C. B. Thompson , “Metabolic Reprogramming: A Cancer Hallmark Even Warburg Did Not Anticipate,” Cancer Cell 21 (2012): 297–308, 10.1016/j.ccr.2012.02.014. PMC3311998 22439925

[43] A. Glaviano , A. S. C. Foo , H. Y. Lam , et al., “PI3K/AKT/mTOR Signaling Transduction Pathway and Targeted Therapies in Cancer,” Molecular Cancer 22 (2023): 138, 10.1186/s12943-023-01827-6. PMC10436543 37596643

[44] R. Kanai , H. Wakimoto , R. L. Martuza , and S. D. Rabkin , “A Novel Oncolytic Herpes Simplex Virus That Synergizes With Phosphoinositide 3‐Kinase/Akt Pathway Inhibitors to Target Glioblastoma Stem Cells,” Clinical Cancer Research 17 (2011): 3686–3696, 10.1158/1078-0432.CCR-10-3142. PMC3107877 21505062

[45] I. R. Cho , S. S. Koh , H. J. Min , et al., “Down‐Regulation of HIF‐1α by Oncolytic Reovirus Infection Independently of VHL and p53,” Cancer Gene Therapy 17 (2010): 365–372, 10.1038/cgt.2009.84. 20075985

[46] I. Yousaf , J. Kaeppler , S. Frost , L. W. Seymour , and E. J. Jacobus , “Attenuation of the Hypoxia Inducible Factor Pathway After Oncolytic Adenovirus Infection Coincides With Decreased Vessel Perfusion,” Cancers 12 (2020): 851, 10.3390/cancers12040851. PMC7225929 32244697

[47] S. Chouaib , Y. Messai , S. Couve , B. Escudier , M. Hasmim , and M. Z. Noman , “Hypoxia Promotes Tumor Growth in Linking Angiogenesis to Immune Escape,” Frontiers in Immunology 3 (2012): 21, 10.3389/fimmu.2012.00021. PMC3341970 22566905

[48] H. Miao , W. Gao , L. Zhong , et al., “VBP1 Promotes Tumor Proliferation as a Part of the Hypoxia‐Related Signature in Esophageal Squamous Cell Carcinoma,” Human Cell 37 (2024): 1141–1155, 10.1007/s13577-024-01068-9. PMC11194215 38700744

[49] L. Yu and C. A. Hales , “Long‐Term Exposure to Hypoxia Inhibits Tumor Progression of Lung Cancer in Rats and Mice,” BMC Cancer 11 (2011): 331, 10.1186/1471-2407-11-331. PMC3199866 21812995

[50] X.‐D. Yang , Y. Cheng , J.‐F. Jia , X.‐S. Cao , and Y. Yang , “Effect of Long‐Term Exposure to Hypoxia on the Proliferation of Colon Cancer Cells *in Vitro* ,” Tumor 33 (2013): 42–47, 10.3781/j.issn.1000-7431.2013.01.007.

[51] J. Druker , J. W. Wilson , F. Child , D. Shakir , T. Fasanya , and S. Rocha , “Role of Hypoxia in the Control of the Cell Cycle,” International Journal of Molecular Sciences 22 (2021): 4874, 10.3390/ijms22094874. PMC8124716 34062959

[52] J. Lü , C. Zhang , J. Han , et al., “Starvation Stress Attenuates the miRNA‐Target Interaction in Suppressing Breast Cancer Cell Proliferation,” BMC Cancer 20 (2020): 627, 10.1186/s12885-020-07118-3. PMC7339532 32631271

[53] K. Izuishi , K. Kato , T. Ogura , T. Kinoshita , and H. Esumi , “Remarkable Tolerance of Tumor Cells to Nutrient Deprivation: Possible New Biochemical Target for Cancer Therapy,” Cancer Research 60 (2000): 6201–6207, https://aacrjournals.org/cancerres/article/60/21/6201/506825/Remarkable‐Tolerance‐of‐Tumor‐Cells‐to‐Nutrient. 11085546

[54] C. Lee , L. Raffaghello , S. Brandhorst , et al., “Fasting Cycles Retard Growth of Tumors and Sensitize a Range of Cancer Cell Types to Chemotherapy,” Science Translational Medicine 4 (2012): 124ra27, 10.1126/scitranslmed.3003293. PMC3608686 22323820

[55] M. Shi , J. Hou , S. Shao , et al., “Short‐Term Starvation Weakens the Efficacy of Cell Cycle Specific Chemotherapy Drugs Through G1 Arrest,” International Journal of Molecular Sciences 24 (2023): 2498, 10.3390/ijms24032498. PMC9917170 36768821

[56] X. Yang , A. Wei , Y. Liu , G. He , Z. Zhou , and Z. Yu , “IGF‐1 Protects Retinal Ganglion Cells From Hypoxia‐Induced Apoptosis by Activating the Erk‐1/2 and Akt Pathways,” Molecular Vision 19 (2013): 1901–1912, https://pmc.ncbi.nlm.nih.gov/articles/PMC3774573/. PMC3774573 24049436

[57] E. Crosas‐Molist , V. Graziani , O. Maiques , et al., “AMPK Is a Mechano‐Metabolic Sensor Linking Cell Adhesion and Mitochondrial Dynamics to Myosin‐Dependent Cell Migration,” Nature Communications 14 (2023): 2740, 10.1038/s41467-023-38292-0. PMC10202939 37217519

[58] L. Li , C. O. Madu , A. Lu , and Y. Lu , “HIF‐1α Promotes a Hypoxia‐Independent Cell Migration,” Open Biology Journal 3 (2010): 8–14, 10.2174/1874196701003010008. PMC2946250 20882121

[59] Q. Wu , L. You , E. Nepovimova , et al., “Hypoxia‐Inducible Factors: Master Regulators of Hypoxic Tumor Immune Escape,” Journal of Hematology & Oncology 15 (2022): 77, 10.1186/s13045-022-01292-6. PMC9166526 35659268

[60] K. Marek , F. Armando , T. Asawapattanakul , et al., “Functional Granulocyte‐Macrophage Colony‐Stimulating Factor (GM‐CSF) Delivered by Canine Histiocytic Sarcoma Cells Persistently Infected With Engineered Attenuated Canine Distemper Virus,” Pathogens 12 (2023): 877, 10.3390/pathogens12070877. PMC10385001 37513724

