# Supplementary material for: Canine Distemper Virus‐Induced Transcriptional Modulation of Canine Histiocytic Sarcoma Cells in an In Vitro Model of Tumour Zones
Source: J Cell Mol Med. 2026 Oct 14;30(20):e71409. doi: 10.1111/jcmm.71409 (PMC13662832; doi:10.1111/jcmm.71409)
Supplement: Supplementary file 1 [file JCMM-30-e71409-s006.pdf]

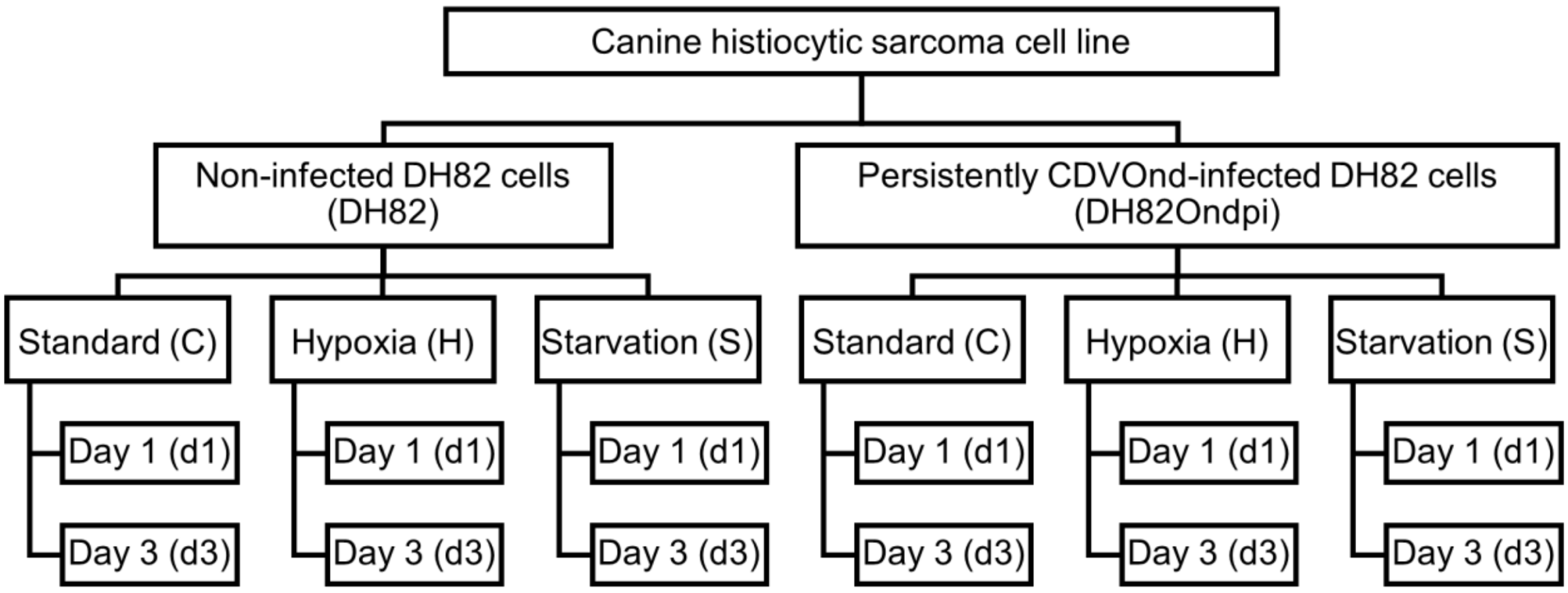

Figure S1 Schematic overview of the experimental design. Canine distemper virus-infected and non-infected canine histiocytic sarcoma cells (DH82 cells) were cultured under three conditions (standard (C), hypoxia (H) and starvation (S)) and harvested at two time points (1 day and 3 days).
